# A Batch-Aware, Subject-Grouped Pipeline to Prioritize HIIT-Associated DNA Methylation Candidates with Cross-Cohort Evaluation in Skeletal Muscle

**DOI:** 10.3390/ijms27198523

**Published:** 2026-09-24

**Authors:** Bruce Mingyeung Song, Kenneth Hon Kim Ban, Caroline Guat Lay Lee, Xavier Wezen Chee

**Affiliations:** 1 Department of Biochemistry, Yong Loo Lin School of Medicine, National University of Singapore, Singapore 117596, Singaporekenneth_ban@nus.edu.sg (K.H.K.B.); bchleec@nus.edu.sg (C.G.L.L.); 2 Cancer and Stem Cell Biology Programme, Duke-NUS Medical School, Singapore 169857, Singapore

**Keywords:** DNA methylation, high-intensity interval training (HIIT), skeletal muscle, methylation candidate prioritization, subject-grouped cross-validation, cross-cohort evaluation

## Abstract

Skeletal muscle DNA methylation is closely associated with molecular adaptation to exercise training, but longitudinal studies in this area are constrained by the difficulty of repeated muscle biopsy sampling and extended follow-up. The resulting datasets are typically small, with repeated measurements within individuals and complex sub-cohort or batch structure. To address these limitations, we developed a methylation candidate-discovery pipeline that integrates batch-aware statistical screening, subject-grouped nested cross-validation, and a binary/multi-class/temporal three-axis consensus screen. We applied this pipeline to the public skeletal-muscle HIIT cohort GSE171140 (195 samples from 52 unique participants; EPIC v1.0; vastus lateralis), in which the 8- and 12-week time points derived from a single sub-cohort. The pipeline estimated per-locus evidence within each sub-cohort, combined these estimates using Fisher’s method, implemented a 5 × 3 nested cross-validation grouped by individual_id, and required candidate CpGs to pass all three task axes. In internal evaluation, LR-L2 achieved AUC = 0.754 (subject-level bootstrap 95% CI [0.689, 0.832]; permutation *p* ≤ 0.002), supported by synthetic-signal recovery and method comparison. The consensus screen reduced 95,477 high-variance CpGs to 21 L6 candidates and one L5 sentinel (cg06639166 near SIM1). In participant-aware mixed-model reanalysis, 20 of the 21 candidates remained associated with training status and 19 of 21 were genome-wide significant after false-discovery-rate correction, with little evidence of sub-cohort heterogeneity in sensitivity analyses. Within the extended MHC (6p21.33), the TNXB five-CpG cluster formed an internally coherent regional signal supported by a 25-CpG DMR (Stouffer *p* = 3.15 × 10^−44^), although genetic, ancestry-related, and copy-number contributions cannot be ruled out on the basis of methylation-only data. We treated external evaluations as bounded checks. Specifically, GSE268211 provided a small partner-laboratory, same-platform, same-tissue consistency check (n = 10; five paired subjects; transfer AUROC 0.96–1.00 with unstable confidence intervals). GSE60655 showed same-tissue endurance-training directional concordance (15/15 testable CpGs sign-concordant, 4/15 raw *p* < 0.05, 0/15 BH-FDR, 34 analytical samples from 17 paired participants). Finally, GSE213363 provided a cross-tissue/cross-disease stress test (AUC = 0.673), in which all 21 candidates were testable, but locus-level concordance was limited in paired analyses. In the paired DNAm-age analysis (15 participants), only Horvath2013 remained significant after multiple-testing correction (mean change +1.40 years, 95% CI +0.56 to +2.23; BH-FDR = 0.015); the other clocks did not. This study provides a reusable analytical framework and a conservative shortlist of exploratory methylation candidates for evaluation in larger, genotype-aware, same-tissue exercise cohorts.

## 1. Introduction

### 1.1. Exercise-Induced Skeletal Muscle Epigenetic Adaptation

DNA methylation is a stable yet reversible epigenetic mark on the mammalian genome. Through cytosine methylation at CpG dinucleotides, this mark contributes to the fine regulation of cell identity, lineage differentiation, and gene expression [1]. Although long regarded as a hallmark of terminal differentiation and stable homeostasis, this mark remains dynamic in mature tissues. Accumulating evidence indicates that adult tissues, despite having completed lineage specification, retain a substantial capacity for dynamic methylation remodeling, undergoing rapid and reversible site-level changes in response to physiological stimuli and environmental exposures [2].

As a prototypical, highly plastic post-mitotic tissue, skeletal muscle is continuously exposed to mechanical loading, energy metabolism, and inflammatory stress. This ongoing exposure makes skeletal muscle an ideal model for studying exercise-induced, tissue-level epigenetic adaptation [3]. Prior studies of skeletal muscle biopsies have documented a global decrease in methylation following training interventions, along with training-duration-dependent, site-level changes at several metabolic, contractile, and myofiber-related loci [4,5]. Together, these findings support a potential role for dynamic DNA methylation remodeling in skeletal muscle adaptation to exercise training [6].

### 1.2. HIIT Specificity and Cardiometabolic Benefits

High-intensity interval training (HIIT) consists of short, near-maximal efforts interspersed with brief recovery periods. Compared with moderate-intensity continuous training (MICT), HIIT achieves comparable or superior physiological adaptation benefits in a shorter training time [7]. Multiple randomized controlled studies and reviews have reported that HIIT significantly improves cardiometabolic indices, including cardiorespiratory fitness, skeletal muscle mitochondrial biogenesis, and insulin sensitivity [8]. These benefits, together with the short time commitment, have contributed to the growing use of HIIT in athletic, health-promotion, and clinical settings.

Skeletal muscle epigenetic studies focusing on HIIT remain scarce. Most existing reports concentrate on either the acute response to a single bout of exercise or short-term interventions lasting only a few weeks; very few incorporate longitudinal sampling across multiple training time points [9]. In this context, the public dataset GSE171140 provides a relatively rich longitudinal methylation resource for HIIT in skeletal muscle. By profiling vastus lateralis biopsies collected at multiple training stages on the Illumina EPIC v1.0 platform, this dataset furnishes a data foundation for the discovery of HIIT-associated sites [10]. Acute responses to a single exercise bout and adaptations to repeated training involve partly distinct methylation dynamics. To characterize the latter, the present study followed methylation changes across a 12-week HIIT program, using biopsies taken at several stages of training.

### 1.3. Methylation Arrays as a Medium for Skeletal Muscle Candidate Discovery

Covering nearly 860,000 CpG sites, the Illumina Infinium MethylationEPIC v1.0 array offers substantially higher density at distal regulatory elements, enhancer regions, and FANTOM5-annotated sites than the previous-generation 450 K array, making it the mainstream platform for human tissue methylation studies [11]. Array-based methylation analysis also carries well-described analytical challenges, including cross-reactive and SNP-affected probes, batch effects, normalization choices, and the cellular heterogeneity of bulk tissue [11,12]. These considerations shaped the preprocessing, probe-level checks, and batch-aware design described in the Methods section. However, skeletal muscle methylation data remain scarce in public databases compared to blood or tumor tissue data, which severely limits opportunities for independent validation in the same tissue across different laboratories or array platforms. To address this gap, the present study uses GSE171140 as a discovery cohort to systematically evaluate site screening and cross-cohort reproducibility in the relatively understudied and data-scarce field of skeletal muscle methylation.

To assess the cross-cohort robustness of candidate sites, this study included three independent evaluation cohorts: GSE268211 (partner laboratory, same EPIC platform, skeletal muscle biopsy); GSE213363 (independent laboratory, resistance and aerobic exercise in women with polycystic ovary syndrome, whole blood); and GSE60655 (previous-generation 450 K array, vastus lateralis, three months of endurance training) [13,14]. Together, these three cohorts provide comprehensive coverage across four dimensions: laboratory, array, tissue, and training modality. Specific protocol parameters are detailed in Section 4.8.

### 1.4. Existing Methodological Limitations

In paired or longitudinal exercise epigenetic studies, subjects often contribute methylation samples at multiple time points. If cross-validation assigns samples at random, multiple time points from the same subject may fall simultaneously into both the training and test sets; the model can then yield inflated accuracy estimates by exploiting unique individual methylation signatures rather than the true exercise response [15]. Although grouping by individual in a GroupKFold split offers a conservative scheme for estimating generalization in paired designs, this approach is not yet standard practice for reporting in the exercise epigenetics literature [16].

Small-sample exercise cohorts often comprise multiple recruitment waves or independent sub-cohorts that are structurally confounded with sex ratio and intervention duration. Performing a pooled *t*-test or an overall limma analysis directly on such pooled samples can cause sub-cohort effects to be erroneously identified as exercise responses. Furthermore, when the batch label is fully confounded with the biological variable, ComBat-type preprocessing batch correction can inadvertently attenuate the true biological signal instead of merely removing technical batch effects [17].

Screening candidate sites based on a single binary contrast via a *t*-test or limma discards valuable information from multi-time-point dose–response relationships and within-subject temporal trajectories [18]. Under conditions of sub-cohort heterogeneity, even candidates that survive multiple-testing correction may be dominated by differences in sub-cohort means rather than genuinely reflecting the training response [19]. Integrating the three axes of binary, multi-class, and temporal analyses into a consensus screen can improve the robustness of site selection; however, this approach remains uncommon in exercise epigenetics research.

Independent cross-cohort evaluation of exercise-associated epigenetic candidate sites remains rare. Most studies rely on a single discovery cohort, and evaluating reproducibility across laboratories, platforms, or disease contexts is not yet standard practice. Relying on statistical significance within a single cohort makes it difficult to rule out cohort-specific spurious signals. Furthermore, differences between array generations impose additional limitations on the scope of site-level comparisons across public datasets.

### 1.5. Study Contributions

To address the four methodological limitations described above, this study proposes a methylation candidate discovery pipeline that integrates batch-aware statistical screening, subject-grouped nested cross-validation, and a binary-multi-class-temporal three-axis consensus screen. We applied this pipeline to the public HIIT skeletal muscle methylation cohort GSE171140. To evaluate this pipeline, we designed a structured evaluation comprising a synthetic injection benchmark, cross-random-seed reproducibility, subject-level bootstrap and permutation testing, pooled out-of-fold model comparison, cross-method comparison against limma and minfi, and evaluation in multiple independent external cohorts.

Beyond analyzing the HIIT skeletal muscle methylation data in this study, this pipeline provides a methodological reference for other high-dimensional, small-sample, multi-time-point precision candidate discovery tasks characterized by batch or sub-cohort structures. Its transferability stems primarily from three reusable designs: estimating evidence within each sub-cohort before combining it, splitting training and test sets at the participant level, and using a multi-task consensus screen to reduce single-contrast bias. Collectively, these designs make the pipeline transferable to other longitudinal methylation studies with small samples, repeated measurements, and sub-cohort structure.

## 2. Results

### 2.1. Pipeline Evaluation

The evaluation protocols described in Section 4.7 and Section 4.8 jointly constitute 13 evidence lines; the overall pipeline schematic is shown in Figure 1A. The complete evaluation portfolio, with the numerical anchor, source analysis, and cross-reference for each line, is summarized in Table S5. Below, we report the key numerical results.

For the binary HIIT vs. control task, an LR-L2 (Ridge) classifier, evaluated using 5-outer × 3-inner subject-grouped nested cross-validation, yielded a mean outer-fold AUC of 0.754 at both the L5 (±0.046) and L6 (±0.042) thresholds (mean ± std across 5 outer folds). Subject-level bootstrap resampling of the 52 independent individuals 1000 times provided a 95% confidence interval of [0.689, 0.832]. Furthermore, a 500-iteration permutation null, which involved within-individual shuffling of binary labels while preserving individual pairing structure, revealed that the observed AUC exceeded the maximum null distribution value (max null AUC = 0.676), with a one-sided *p* ≤ 0.002. Figure 2A displays the ROC curves with per-fold 95% CI bands, and Figure 2B shows the permutation null histogram with the observed AUC position annotated.

Following the synthetic injection protocol described in Section 4.7.1, we observed two main outcomes. (a) For single-axis binary injection (d ∈ {0.3, 0.5, 0.7, 1.0} × 3 random seeds), the TPR at the L6 threshold increased with effect size to 0.64, 0.87, 0.98, and 1.00, respectively (mean TPR = 0.87 at d = 0.5). (b) Conversely, coordinated three-axis injection at d = 0.8 yielded a coordinated TPR of only 0.018. This resulted in the intersection set shrinking approximately 325× (from roughly 45,000 binary L6 candidates to approximately 140 intersection candidates), while the injection-defined FDR remained high due to single-axis-only spillover and the null pool. These findings suggest that the three-axis intersection serves as a conservative prioritization procedure, yielding a small but stable shortlist, rather than a high-sensitivity recovery scheme. The temporal cutoff (|slope| > 0.003 and R^2^ > 0.15) acts as the primary bottleneck for stringency. Complete TPR and injection-defined FDR values are provided in Table S1. The three-axis intersection therefore represents a deliberate sensitivity–specificity trade-off. The candidate set is accordingly interpreted as a shortlist of CpGs with consistent behavior across the three task axes, whereas single-axis screening retains sensitivity for broader discovery.

Following the protocol in Section 4.7.2, the entire pipeline was run independently using three random seeds (42, 123, and 2024). The L5 shortlist (1 CpG, cg06639166) and the L6 shortlist (21 CpG) generated by all three seeds were completely identical, demonstrating a pairwise Jaccard index of 1.0. This determinism indicates that the pipeline introduced no randomness at the implementation level; robustness to sampling variation was assessed separately by the participant-aware reanalysis (Section 2.3).

Following the protocol in Section 4.7.4, which involved recomputing per-fold rankings using only train subjects, the method-comparison K-curve was evaluated across a 5 K × 3 method × 5 fold matrix. The pipeline binary method yielded AUC values of 0.658, 0.661, 0.697, 0.718, and 0.712 for K = 21, 50, 100, 500, and 1000, respectively. Limma produced values of 0.635, 0.661, 0.668, 0.706, and 0.706 for the same K values, while minfi achieved 0.698, 0.686, 0.686, 0.713, and 0.716. The pipeline performed no lower than limma for four out of five K values (K = 50 was a tie) and was the highest among the three methods within the K = 100–500 “sweet-spot” interval. At K = 21, which represents the pipeline’s native shortlist size, minfi (0.698) was marginally higher than the pipeline (0.658); all three methods were closely aligned. This result is reported faithfully in the main text, reflecting the original comparison without selective emphasis. Figure 2D illustrates the three-method K-curve with error bands.

For ElasticNet (l1_ratio = 0.9), trained with an expanded C grid {0.001, 0.01, 0.1, 1, 10, 100, 1000}, the optimal C values across the five outer folds were distributed as 1 × 10, 3 × 100, and 1 × 1000 (4/5 folds at 100 or higher). This model exhibited a sparsity of only 0–19% (near-zero sparsity in each fold), and its baseline AUC of 0.755 +/− 0.039 was consistent with LR-L2. This low sparsity (0–19%) indicates that the binary signal was distributed across hundreds of CpGs, making it difficult to represent adequately with a sparse, compact feature set of 5–21 CpGs. This finding provides key evidence for a distributed signal, which is further corroborated by the cross-model top-100 feature Jaccard of ≈0.13 (Section 2.2).

### 2.2. Binary HIIT Classification Performance

For the binary HIIT vs. control task, the internal CV AUCs of the five model families, evaluated under the framework of Section 4.5, were as follows: LR-L1 = 0.679 ± 0.066; LR-L2 = 0.754 ± 0.042 (best); RF = 0.727 ± 0.040; ElasticNet = 0.755 ± 0.039; and LightGBM = 0.732 ± 0.063. Ridge, RandomForest, ElasticNet, and LightGBM clustered on a 0.73–0.76 plateau, while LR-L1 was notably lower (0.679). This lower performance of LR-L1 was consistent with a sensitivity analysis, which revealed the L1 + Liblinear configuration to be suboptimal; replacing the Liblinear solver with Saga yielded significantly higher AUCs for LR-L1 (0.748 at L5/0.758 at L6 vs. Liblinear’s 0.673/0.679). Results were essentially unchanged when the variance filter was refit within each outer training fold (LR-L2 AUC 0.765 ± 0.061; Section 4.7.5). Furthermore, the mean pairwise Jaccard index of the top-100 important-feature lists across the five models was 0.13 (LR-L1 ∩ LR-L2 = 0.344; LR-L1 ∩ RF = 0.026; LR-L2 ∩ RF = 0.025; three-way intersection = 0.015). This low Jaccard index indicates that different models achieved their performance plateau in distinct feature subspaces, corroborating that the binary signal was distributed rather than concentrated in a compact feature set.

To compare model families while accounting for the shared training data across outer folds, out-of-fold predictions were pooled and resampled by participant (2000 bootstrap replicates; both penalized logistic models fitted with the Saga solver, consistent with the solver sensitivity noted above). Pooled AUROCs were 0.771 (95% CI 0.696–0.835) for LR-L1, 0.758 (0.689–0.821) for LR-L2, 0.745 (0.670–0.813) for ElasticNet, 0.722 (0.666–0.774) for RF, and 0.695 (0.629–0.750) for LightGBM (Figure 2C). The five confidence intervals overlapped substantially, and the only paired differences excluding zero favored the two logistic models over LightGBM (bootstrap *p* = 0.005 and *p* = 0.023); LR-L2 and LR-L1 were statistically indistinguishable (*p* = 0.225). This pattern is consistent with a signal distributed across many CpGs rather than concentrated in a model-specific feature set.

For the multi-class five-way task (Control/Baseline/4 W/8 W/12 W), LR-L2 at the L6 threshold yielded a macro-OVR mean AUC of 0.760 ± 0.029. The temporal four-class ordinal task (HIIT intervention 0 W/4 W/8 W/12 W) achieved a macro-OVR mean AUC of 0.736 ± 0.026 with a Spearman ρ = 0.334. The multi-class confusion matrix (Supplementary Figure S1) revealed a relatively high misclassification rate between the control and baseline labels, suggesting a genuine epigenetic overlap between these two classes in the cohort. Additionally, 12W was primarily misclassified as 4W. This misclassification pattern is related to the study design, where the 8W and 12W time points were present only in the Inter2 sub-cohort. This residual confounding warrants further explanation (see Section 3.7).

### 2.3. The Corrected 21-CpG Shortlist

The pipeline funnel (Figure 1B,C) began with 865,859 raw CpGs on the EPIC v1.0 platform. After preprocessing, 368,596 CpGs were retained. Variance-based top-k pre-filtering further reduced this set to 95,477 CpGs. Subsequent per-axis L6 batch-aware single-pass whole-cohort screening identified 12,978 CpGs for the binary task, 705 for the multi-class task, and 30 for the temporal task. The intersection of these three axes then yielded a 21-CpG L6 shortlist (Figure 3A). Applying the stricter L5 threshold (|d| > 0.30 ∩ F > 4.0 ∩ R^2^ > 0.20) further contracted this shortlist to a single CpG (cg06639166), which passed all four strict criteria simultaneously. Figure 1B illustrates the staged funnel diagram (CpG count by stage), and Figure 1C presents the three-axis Venn diagram, showing the binary, multi-class, and temporal axes along with their intersection region.

Of 21 CpGs, 17 were located within or near named gene bodies. These included a five-CpG cluster in TNXB (cg02740950, cg05483184, cg09996288, cg17001689, cg26997880), all situated within the chr6:32,055,370–32,055,534 165 bp Island Body region. Additional gene-associated CpGs were JARID2, FERMT3, MYO16, NIBAN2, MARCHF2, CTC1, LDLRAD4, PIWIL4, RASGRP3, PITX1-AS1, LINC01973, and LINC01111 (using their current HGNC symbols). The remaining four CpGs resides in intergenic or unannotated segments, including cg06639166 (chr6:100,913,923), which was intergenic and approximately 1 kb upstream of the SIM1 TSS.

ChromHMM 15-state annotation from Roadmap E108 (female skeletal muscle) classified all five TNXB CpGs as 14_ReprPCWk (weak polycomb-repressed). Furthermore, cg06639166 was annotated as 13_ReprPC (polycomb-repressed, not a FANTOM5 enhancer), and cg00075402 as 15_Quies (quiescent). Approximately 33% (7/21) of the CpGs fall into active-chromatin categories (active enhancer or TssAFlnk) [20].

EWAS Catalog queries revealed that 19/21 (90%) of these CpGs had a prior published association. Of these, 35 entries were aging-related, with no exercise-specific hits. This indicates that the majority of the 21 CpGs have been previously reported in the aging or metabolic-phenotype literature.

Table 1 lists all annotation fields for the 21 CpGs, and Supplementary Figure S2 shows their chromosomal distribution.

Region indicates the UCSC RefGene group (TSS1500/TSS200/5′UTR/1stExon/Body/3′UTR) and the CpG-island relation: Island; N_Shore/S_Shore (±2 kb from an island); N_Shelf/S_Shelf (±2 kb from the shores); or OpenSea (no relation). ChromHMM codes (Roadmap 15-state) are defined as follows: 13_ReprPC/14_ReprPCWk (weak polycomb-repressed); 15_Quies (quiescent); EnhBiv (bivalent enhancer); and TssAFlnk (flanking active TSS). The sample size was n = 195 (HIIT 102; control 93); gene symbols use current HGNC nomenclature. q (screen), Benjamini–Hochberg false-discovery rate of the Fisher-combined binary-axis *p* value across all 95,477 screened CpGs; q (mixed model), Benjamini–Hochberg false-discovery rate across all 368,596 preprocessed CpGs from a linear mixed model of β values with a participant random intercept, adjusted for sub-cohort and sex (Section 4.3). Corresponding screen-level FDRs on the multi-class and temporal axes did not fall below 0.50 for any candidate. † cg20321058 did not remain significant in the participant-aware mixed-model reanalysis.

To account for repeated sampling of the same participants, all 21 candidates were re-examined with linear mixed models (participant random intercept, adjusted for sub-cohort and sex; Section 4.3). In total, 20 of the 21 CpGs remained associated with training status on both the β and M scales (BH-FDR < 0.05 across the candidates), and 19 of 21 also fell below the 5% BH-FDR threshold when correction was applied across all 368,596 analyzed CpGs (Table 1). Effect directions matched the screening estimates at all 21 sites, and the five TNXB CpGs ranked among the 100 smallest mixed-model *p* values genome-wide. The exception was cg20321058 (RASGRP3), which was not significant in any mixed-model specification (*p* = 0.235) and is flagged accordingly in Table 1.

DMRcate, using EPICv1 mode and a Stouffer *p* < 0.05, identified 3540 DMRs in the HIIT vs. control comparison. The 21 CpGs mapped to 13 unique named genes, with 8 (62%) of these genes overlapping a DMR: TNXB, NIBAN2, PIWIL4, PITX1-AS1, FERMT3, LINC01973, MARCHF2, and JARID2 [21].

Key top DMRs included the TNXB region at chr6:32,054,561–32,056,249 (25 CpG/1.7 kb/Stouffer *p* = 3.15 × 10^−44^/HMFDR = 4.22 × 10^−4^). This DMR provided same-dataset regional corroboration of the TNXB 5-CpG cluster.

Two additional key DMRs were found in SIM1: chr6:100,903,561–100,906,895 (17 CpG, Stouffer *p* = 8.25 × 10^−36^, HMFDR = 9.05 × 10^−4^) and chr6:100,894,513–100,897,445 (15 CpG). Both SIM1 DMRs are located within the SIM1 gene body, approximately 7 kb and 16 kb from cg06639166, respectively. Given that cg06639166 itself is approximately 1 kb upstream of the SIM1 TSS, these findings suggest that cg06639166 lies within a broader regional methylation signal near SIM1.

Table S2 lists the detailed fields of the top 30 DMRs. Region detection was insensitive to covariate adjustment; with sub-cohort and sex in the design matrix, 99.5% of regions were recovered, the TNXB and both SIM1 DMRs were identified at identical coordinates, and 3539 of the 3541 regions also satisfied a harmonic-mean FDR below 0.05 (Section 4.10).

In the participant-aware regional sensitivity analysis, DMRcate 2.16.1 detected 14,666 regions, of which 13,842 met native HMFDR < 0.05; 13 of the 21 candidate CpGs lay within these regions. All four focus regions were recovered: TNXB (chr6:32,053,600–32,056,820; 28 CpGs; native HMFDR = 2.09 × 10^−5^), SIM1 body 1 (chr6:100,903,561–100,906,895; 17 CpGs; 5.10 × 10^−4^), SIM1 body 2 (chr6:100,893,665–100,897,445; 16 CpGs; 5.35 × 10^−3^), and the region containing cg06639166 (chr6:100,913,923–100,915,805; 8 CpGs; 6.24 × 10^−4^). Each recovered region covered its original interval; the TNXB and second SIM1-body boundaries expanded, whereas the other two were unchanged. All four had negative mean adjusted Δβ (Table S2, additional panel), supporting the regional signal after accounting for repeated observations within participants.

### 2.4. Candidate Case Studies

A five-CpG cluster within TNXB (chr6:32,055,370–32,055,534, 165 bp Island Body) was identified. All five sites within this cluster—cg02740950 (d = −0.717, *p* = 1.88 × 10^−4^), cg05483184 (d = −0.688, *p* = 3.62 × 10^−4^), cg09996288 (d = −0.650, *p* = 5.05 × 10^−4^), cg17001689 (d = −0.723, *p* = 1.30 × 10^−4^), and cg26997880 (d = −0.644, *p* = 7.13 × 10^−4^)—were hypomethylated, with an averaged Cohen’s d range of −0.65 to −0.72. Of these, 4/5 sites had a prior association with smoking or aging in the EWAS Catalog, but none had prior exercise associations. Furthermore, DMRcate identified a region-level differentially methylated region (DMR) within TNXB (chr6:32,054,561–32,056,249; 25 CpGs, 1.7 kb; Stouffer *p* = 3.15 × 10^−44^, HMFDR = 4.22 × 10^−4^). This DMR showed downregulation across the entire segment, encompassing both the five-CpG cluster and an extended segment, thereby providing dual region-level support for the single-CpG signals. None of the 21 candidates, including the five TNXB CpGs, appears on published lists of cross-reactive or SNP-affected EPIC probes. The five TNXB CpGs were strongly co-methylated (pairwise Pearson r = 0.92–0.96, both overall and within each group), consistent with one coherent regional signal rather than five independent candidates. A cross-array, same-tissue evaluation using the GSE60655 dataset (endurance training, vastus lateralis, 450 K, 34 analytical samples from 17 paired participants) revealed that all 5/5 testable TNXB CpGs were concordant with the discovery direction. Of these, 2/5 reached nominal significance (cg05483184 *p* = 0.023; cg09996288 *p* = 0.040), and one additional site was borderline significant (cg02740950 *p* = 0.051). These findings provide cross-array, cross-intervention-modality, and same-tissue directional-concordance support for this region, though they do not constitute definitive biological validation. Roadmap E107 (male skeletal muscle) annotates some sites in the TNXB cluster as EnhBiv (bivalent enhancer), which differs from the E108 (female) annotation of 14_ReprPCWk across the entire segment. This suggests a sex difference in chromatin context within muscle. Figure 4A–C illustrates the boxplots per study group, the DMR browser track, and the GSE60655 forest plot. The biological interpretation of TNXB (tenascin-X, an ECM glycoprotein) is detailed in Section 3.2.

**Figure 3:**
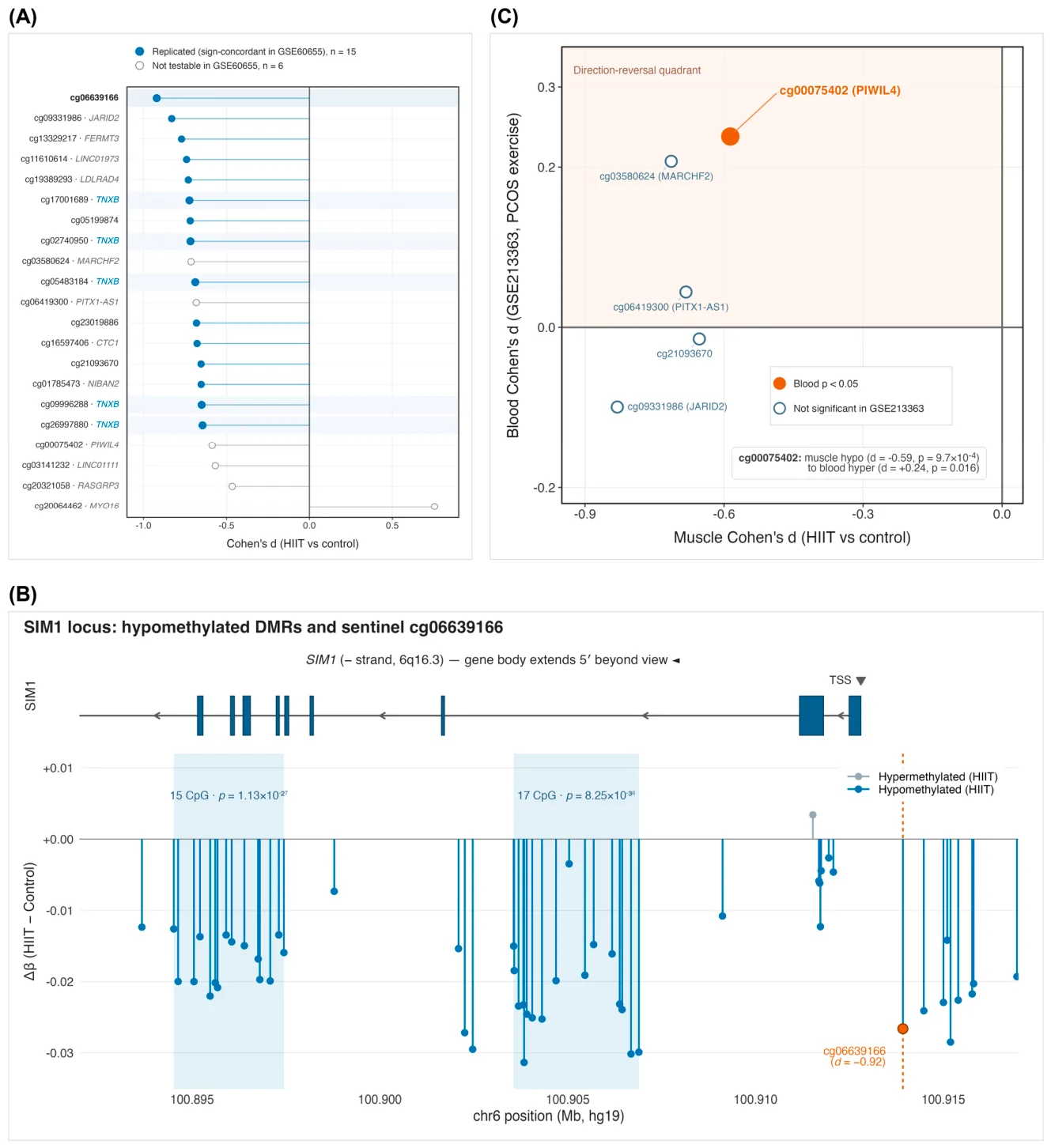
The 21-CpG shortlist and the two single-CpG sentinel loci. (**A**) Forest plot of the 21 L6 candidate CpGs (Cohen’s d, HIIT vs. control, +/− 95% CI); cg06639166 highlighted. (**B**) UCSC Genome Browser view (hg19) of the SIM1 region: cg06639166 approximately 1 kb upstream of the SIM1 TSS and the two SIM1-body DMRs. (**C**) Cross-disease comparison of cg00075402 (PIWIL4): HIIT skeletal-muscle hypomethylation (GSE171140) versus PCOS whole blood (GSE213363). This association is nominal (raw *p* = 0.016; BH-FDR = 0.17) and direction-reversed, indicating no directional replication. DMR, differentially methylated region.

cg06639166 (chr6:100,913,923, intergenic, N_Shore) exhibited the largest effect size among the 21 L6 CpGs (Cohen’s d = −0.921, binary *p* = 5.24 × 10^−6^, F = 6.04, R2 = 0.200). It was also the only CpG that passed the four strict L5 criteria. This CpG is located approximately 1 kb upstream of the SIM1 (single-minded homolog 1) transcription start site (TSS). SIM1 is a key monogenic obesity gene, associated with hyperphagia and early-onset severe obesity, and functions as a hypothalamic neurodevelopmental transcription factor involved in the melanocortin signaling pathway [22]. DMRcate identified two significant differentially methylated regions (DMRs) near SIM1 (17 CpG and 15 CpG), which is consistent with a regional methylation signal near SIM1 that includes cg06639166. Cross-cohort evaluation showed directional concordance in the same-tissue 450 K GSE60655 cohort (raw *p* = 0.159, non-significant, 34 analytical samples from 17 paired participants; power-limited; see Section 3.7). However, no significant association was found in the GSE213363 PCOS whole blood dataset. Roadmap E108 annotates cg06639166 as 13_ReprPC (polycomb-repressed) and not a FANTOM5 enhancer. SIM1 exhibits a median TPM < 1 in GTEx vastus lateralis, indicating low expression within muscle. Therefore, the observed methylation change at cg06639166 in muscle may reflect a long-range regulatory imprint of SIM1 rather than a direct marker of muscle-local SIM1 transcription. The EWAS Catalog records six prior aging-related associations for this site, but no prior exercise or muscle-related associations. Figure 3A (highlighting cg06639166 within the 21-CpG forest) and Figure 3B present a UCSC browser snapshot of the SIM1 region. The distribution of this site by study group is shown in Supplementary Figure S3.

cg00075402 (chr11:94,300,101, PIWIL4 TSS1500, OpenSea, EPIC-only) has not been reported previously. It has no PubMed mentions, is not a member of any of the 0/5 published epigenetic clocks [23,24,25,26,27], and has no prior hits in the EWAS Catalog. The pipeline output for this CpG showed an averaged Cohen’s d = −0.586, binary *p* = 9.73 × 10^−4^, F = 3.91, and R2 = 0.200, meeting the L6 criteria. In GSE213363, where all 21 shortlist CpGs were testable, cg00075402 showed no association in the paired analysis (Wilcoxon *p* = 0.18; Table S7); the nominal, direction-reversed association observed in the unpaired screen (raw *p* = 0.016) did not survive correction across the testable candidates (BH-FDR = 0.17). This site therefore currently lacks external support and remains an exploratory candidate. As cg00075402 is EPIC-only and absent from the 450 K array, it could not be tested in GSE60655. PIWIL4 (P-element-induced wimpy testis-like 4) encodes a core protein of the Argonaute-family piRNA pathway, which is involved in germline and somatic stem cell maintenance and transposon silencing. Muscle expression of PIWIL4 is low (GTEx vastus lateralis median TPM < 1) [28]. The observed change at this site may therefore reflect an epigenetic imprint of the piRNA pathway in muscle in response to exercise training. Small RNA-seq and matched expression data are needed to test this. Figure 3C illustrates the cross-disease comparison panel, highlighting the nominal but direction-reversed feature of cg00075402.

Important caveats apply to the three candidate loci. First, the five TNXB CpGs are located within chr6p21.33/the extended MHC, a polymorphic, mQTL-rich, and CNV-prone region that includes the RCCX module. Publicly available methylation data for this region lack matched genotype, ancestry, and CNV information. Probe-level risks were assessed against published lists of cross-reactive and SNP-affected EPIC probes, and none of the 21 candidates was flagged; contributions from mQTLs/genetics, ancestry/population stratification, and copy number variations, however, cannot be excluded (Supplementary Table S4). Furthermore, the TNXB cluster exhibits sex-related chromatin-state differences (EnhBiv in Roadmap E107 male skeletal muscle versus 14_ReprPCWk across the segment in E108 female skeletal muscle), which restricts a sex-neutral, exercise-specific interpretation. Second, cg06639166 is polycomb-repressed (13_ReprPC) rather than an active enhancer in muscle, so mechanistic interpretation should be cautious. Third, cg00075402 shows a reversed direction between HIIT muscle hypomethylation and PCOS whole-blood post-training hypermethylation, suggesting context-dependent regulation across site, tissue, and disease. Given these caveats, the 21 CpGs should be considered discovery candidates, awaiting matched transcriptomic, genotype-aware, and functional follow-up, rather than confirmed mechanistic biomarkers.

### 2.5. Cross-Cohort Evaluation

External transfer results for GSE268211 (partner-laboratory EPIC v1.0 vastus lateralis, n = 10, 5 paired baseline-vs-8W HIIT), obtained under the protocol described in Section 4.8.1, demonstrated transfer AUROC of 0.96–1.00 across the five fitted models, with wide or degenerate bootstrap confidence intervals and accuracy of 0.50–0.90 (Table S6). These findings indicate directionally consistent transfer within a cohort independent of feature screening and model fitting. However, several limitations were observed: the cohort comprised only 10 samples (five paired subjects), confidence-interval stability was limited, external accuracy varied across models, and the external AUC exceeded the honest internal subject-grouped CV AUC. Consequently, we consider GSE268211 a small, partner-laboratory/same-platform/same-tissue consistency check rather than an adequately powered external validation. Table 2 summarizes these external transfer results.

The discovery analysis employed LR-L2 subject-grouped 5-outer × 3-inner nested cross-validation. For GSE268211, five-model external transfer was performed, from a partner laboratory; however, given its small sample size (10 samples from five paired subjects) and limited confidence-interval and accuracy stability, it should be considered a small-cohort consistency check rather than direct validation evidence. Both GSE213363 and GSE60655 report five-fold subject-grouped cross-validation. Where applicable, per-CpG raw Wilcoxon paired *p* < 0.05 and BH-FDR values are reported. In GSE213363, all 21 shortlist CpGs were testable; per-CpG results are from paired Wilcoxon signed-rank tests across the 56 pre–post pairs (Table S7). For GSE60655, 34 analytical samples represent 17 paired participants (baseline vs. 3-month post), and none of the 15 testable CpGs survived FDR correction. Definitions: EPIC, Infinium Methylation EPIC v1.0 BeadChip; 450 K, Illumina Human Methylation450 BeadChip; PCOS, polycystic ovary syndrome.

Following the protocol detailed in Section 4.8.2, GSE213363, a dataset comprising polycystic ovary syndrome (PCOS) women (EPIC whole blood, n = 112, 56 paired pre-vs-post resistance + aerobic training), was analyzed using five-fold subject-grouped cross-validation. This involved train-only feature screening within each fold and LR-L2 fitting, yielding a mean AUC = 0.673 +/− 0.065. Per-fold AUC values were 0.701, 0.703, 0.744, 0.579, and 0.636, with a range of 0.58–0.74. This modest cross-disease generalization is attributed to differences at three levels: (i) muscle vs. whole-blood tissue specificity, (ii) the HIIT vs. resistance + aerobic intervention-modality difference, and (iii) the influence of the PCOS metabolic background. All 21 shortlist CpGs were testable in GSE213363. In paired analyses across the 56 pre–post pairs, one CpG (cg20064462) fell below the BH-FDR threshold in the direction observed in the discovery cohort, and the five TNXB CpGs showed no change (|Δβ| ≤ 0.002; Table S7). Overall direction concordance (10 of 21) was comparable to that expected for unrelated probes, and no locus-level transfer of the discovery pattern was observed. Table 2 summarizes the per-fold/per-model external evaluation results.

Following the protocol stated in Section 4.8.3, GSE60655 (Nitert/Lindholm endurance training, Illumina 450 K, 34 analytical samples from 17 paired participants with baseline and 3-month post-training measurements, vastus lateralis, same tissue) was analyzed using five-fold subject-grouped cross-validation. This yielded a mean AUC = 0.876 (within-cohort cross-validation), with per-fold values of 1.000, 0.940, 1.000, 0.667, and 0.778. As summarized in Table 2, all 15 testable CpGs showed sign concordance with the discovery direction, indicating same-tissue exercise-associated directional concordance across array platform and training modality. However, this cohort investigated endurance training rather than HIIT, and none of the 15 CpGs survived BH-FDR correction. Therefore, the observed support was directional and exercise-general, but it does not represent interval-training-specific validation. Within the TNXB cluster, all five testable CpGs showed sign concordance, with two of the five being nominally significant, and one TNXB site (cg02740950) was borderline (*p* = 0.051). Figure 4C presents the per-CpG forest, and Figure 5 displays the heatmap. Per-CpG statistics and paired effect sizes are provided in Table S8. The observed sign concordance should be read against a strong cohort-wide pattern; 93% of all probes hypomethylated in the discovery cohort were also hypomethylated after endurance training in GSE60655, and conditional on this pattern, 15 of 15 concordant candidates were unremarkable (matched-background *p* = 0.21);. A within-participant sign-flip test nevertheless indicated a coordinated training response across the panel (*p* = 0.042). Collapsing the five TNXB CpGs into a single cluster-level unit gave consistent results (sign-flip *p* = 0.051; direction-matched background *p* = 0.31).

### 2.6. Sensitivity

To improve experimental precision, we performed a complete subject-grouped nested-CV rerun exclusively in male samples. This was prompted by the fact that all 8W/12W time points originated from the male Inter2 sub-cohort, while baseline/4W time points included mixed-sex samples. The female samples were not excluded due to quality or outlier reasons; they remained part of the full-cohort and sex-covariate analyses. The male-only rerun served solely as a sensitivity analysis. This rerun (n = 149) yielded L5 AUC = 0.769 ± 0.065 and L6 AUC = 0.774 ± 0.067. Both of these threshold levels were comparable to or slightly higher than the full-cohort baseline AUC values of 0.754 ± 0.046 (L5) and 0.754 ± 0.042 (L6), representing a Δ of +0.015 to +0.020. The comparable baseline AUC in the male-only cohort suggested that the binary HIIT vs. control signal was broadly consistent after restriction to male participants in this sensitivity analysis.

When the LR-L2 model was rerun on all 195 samples with sex explicitly added as a covariate (an extended model including a sex term), the results were L5 AUC = 0.747 ± 0.040 and L6 AUC = 0.745 ± 0.037. These values represent a Δ relative to baseline of −0.007/−0.009, which is near zero. The mean sex-term coefficient across the five folds ranged from +0.007 to +0.009, indicating a near-zero logit contribution. This finding was broadly consistent with the male-only sensitivity analysis, and adding sex as a covariate produced little change in the baseline AUC. Together, these sensitivity analyses of sex suggest that the principal findings were robust to adjustment for sex and broadly consistent in the male-only analysis.

The batch-aware Fisher combination (Section 4.3) reduced sensitivity to differences among the three sub-cohorts (Main, Inter1, Inter2) during statistical screening. This was achieved through stratified per-study group screening, using study_group as a stratification factor. Additionally, the nested cross-validation (CV) described in Section 4.4, implemented via GroupKFold by individual_id, ensured that samples from the same subject were not distributed across different folds. Despite these measures, the three-way confounding involving sub-cohorts × sex × time point was only partially mitigated by the batch-aware tests and subject-grouped CV. Residual confounding persisted because the 8W and 12W time points appeared exclusively in Inter2, and because study_group, sex, and time point were not fully separable. Specifically, Main and Inter2 sub-cohorts consisted entirely of male samples, while Inter1 included both male and female samples. The 8W and 12W time points were observed only within the Inter2 sub-cohort. This residual confounding is evident in the multi-class confusion matrix, where 12W samples were primarily misclassified as 4W, reflecting the sub-cohort residual associated with the Inter2-only time points. This limitation is further discussed in Section 3.7. To quantify this underlying structure, a PERMANOVA-style analysis of the discovery feature space was conducted. This analysis revealed that individual identity accounted for the largest share of multivariate variance (R^2^ = 0.624), followed by study_group/sub-cohort (R^2^ = 0.195). In contrast, the five-class time label accounted for a smaller fraction (R^2^ = 0.041; Supplementary Table S3). Consistently, decomposition of the leading principal components (PCs) indicated that variation along PC1 was primarily associated with study_group (88.9%), while variation along PC2 was mainly associated with individual identity (65.5%). This pattern aligns with the decision to employ subject-grouped CV and batch-aware screening. However, it is not sufficient to support the previous interpretation of the temporal-task results as a direct training-duration trajectory.

To separate training effects from sub-cohort membership, the 21 candidates were additionally examined in two participant-aware analyses (Section 4.3): a longitudinal model restricted to Inter2, where all four time points were available, and a common baseline-to-4-week model across the three sub-cohorts with a training-by-sub-cohort interaction term. Fifteen of 21 CpGs showed a significant time effect within Inter2 alone, and 19 of 21 showed a significant common baseline-to-4-week effect (BH-FDR < 0.05). Only one CpG showed a significant interaction on the β scale (none on the M scale): cg20321058, whose heterogeneity was driven by the main sub-cohort rather than Inter2. Sixteen of 21 CpGs changed in the same direction in all three sub-cohorts, and effect estimates correlated across sub-cohorts (r = 0.67–0.72). These analyses provide no evidence that the candidate signal reflects the sub-cohort structure, although the restriction of the 8- and 12-week time points to Inter2 remains a design limitation.

### 2.7. DNAm Age Clocks

Because the HIIT and control samples largely derived from the same participants, the whole-cohort comparison is reported descriptively, and the within-participant paired analysis is treated as the primary analysis. The five epigenetic age clocks showed varying DNAmAge acceleration between the cross-sectional HIIT (n = 102) and control (n = 93) groups (Δ = HIIT − Control). For the four chronological clocks, Δ was measured in years, while for DunedinPACE, it was in pace units. All four chronological clocks showed higher age acceleration in the HIIT samples (Δ ≈ +0.8 to +1.2 years), whereas DunedinPACE was unchanged.

Figure 6A shows the bar chart of Δ acceleration with CI for the five clocks.

The within-subject paired analysis was recomputed using 15 paired subjects (30 samples forming 15 Inter2 baseline-to-12-week pairs), with the subject, rather than the sample pair, serving as the statistical unit. In this primary age-acceleration analysis, all four chronological clocks showed positive mean changes. However, after Benjamini–Hochberg correction across the five clocks, only Horvath2013 remained significant (Δ = +1.40 yr, 95% CI +0.56 to +2.23; paired t *p* = 0.003, BH-FDR = 0.015). Horvath2018 SkinBlood (Δ = +0.75 yr; BH-FDR = 0.070) and PhenoAge (Δ = +1.61 yr; BH-FDR = 0.070) were nominal only. Hannum2013 was not significant (Δ = +0.46 yr; BH-FDR = 0.211), and DunedinPACE was null (Δ = −0.009 pace units; BH-FDR = 0.548).

The same significance pattern was observed on the raw clock-value scale. Thus, the paired analysis should be interpreted as directionally consistent with the cross-sectional comparison, but it is exploratory and FDR-significant for one clock only, rather than serving as independent confirmation across multiple clocks. Figure 6B shows the paired Δ (12W − baseline) of the five clocks with 95% CI, faceted by unit.

Collectively, the cross-sectional clock pattern and the corrected paired analysis suggest a tissue-specific training-associated methylation response in skeletal muscle, rather than evidence of systemic biological aging acceleration. The paired analysis provides secondary, exploratory support for this interpretation, given its limitation to 15 paired subjects. This finding contrasts with the literature on blood-based analysis of the effects of long-term physical activity, where most reviews report DNAmAge deceleration in blood for active individuals [29,30]. This discrepancy reflects a fundamental difference in stress-response biology between muscle and blood. Consequently, the direction of a chronological clock cannot be directly used to infer whether exercise accelerates or slows aging. Therefore, the results of this section are best interpreted as describing the training-associated epigenetic response in muscle to 12 weeks of HIIT, rather than supporting any claim about lifespan or systemic aging. Notably, 4/5 chronological clocks were primarily trained on blood or pan-tissue data; thus, their application to muscle may have amplified the tissue-specific stress signal within the chronological-proxy channel. In contrast, DunedinPACE was trained on longitudinal phenotypic and biomarker phenotypes [24], which may make it relatively robust to tissue-specific noise in the training-associated context. Further discussion is provided in Section 3.4.

## 3. Discussion

### 3.1. Methodological Contribution

This study’s principal methodological contribution is a reusable pipeline for methylation candidate discovery that integrates batch-aware screening, subject-grouped cross-validation, multi-task consensus screening, and multi-layer evaluation. Instead of directly estimating differential methylation in a pooled sample set, the pipeline first estimates CpG-level statistical evidence separately within each study group (Main, Inter1, and Inter2). It then combines *p*-values using Fisher’s method and averages the effect sizes. This design emphasizes screening results that are consistent across sub-cohorts and, when combined with participant-aware mixed-model inference at the locus level (Section 4.3), it is applicable to longitudinal intervention data with small samples, multiple batches, or a pronounced sub-cohort structure.

The second key aspect of the design is subject-grouped 5-outer × 3-inner nested cross-validation, with grouping performed by individual_id. Since subjects can contribute multiple time-point samples in this study, subject-level grouping preserves their independence between the training and test sets during model evaluation, which provides a more conservative generalization estimate. The third feature of the design involves a three-axis consensus screen across binary, multi-class, and temporal axes. A CpG enters the final shortlist only if it simultaneously meets thresholds from three perspectives: intervention state, time-point classification, and temporal trend. This design trades candidate count and sensitivity for a candidate set emphasizing cross-task consistency.

The evaluation results support the stability of the pipeline’s results with this study’s data. Specifically, the internal binary AUC was 0.754 ± 0.046, the subject-level bootstrap 95% CI was [0.689, 0.832], and the permutation test yielded *p* ≤ 0.002. Pooled out-of-fold comparison with participant-clustered resampling showed the model families to be statistically indistinguishable, except that both logistic models outperformed LightGBM (Section 2.2). The three random seeds produced identical outputs (Jaccard = 1.0), reflecting implementation-level determinism, and the K-curve indicated that the pipeline performed well in the K = 100–500 range. The low sparsity of ElasticNet, combined with a cross-model Jaccard of 0.13, suggests that the binary signal is distributed across many CpGs and is not driven by a few single points.

This pipeline’s transferability stems from its design principles, not from any single algorithm. Batch-aware screening, subject-grouped CV, and multi-axis consensus are all applicable to other small-sample, high-dimensional, paired, or multi-time-point candidate discovery tasks. However, this transferability requires re-evaluation for specific data types, tissue contexts, and study designs. Accordingly, this study provides a systematically evaluated analytical framework with particular relevance to small-sample, high-dimensional longitudinal studies involving repeated measurements and structured sub-cohorts.

### 3.2. Interpretive Value of the TNXB-Related Signal

The TNXB region emerged directly from the data-driven candidate-prioritization process as one of the most coherent regional signals in this study. According to the analysis, 5 of the 21 L6 CpGs were located within a 165-bp segment of TNXB and showed consistent HIIT-associated hypomethylation. These CpGs were strongly co-methylated and were encompassed by a 25-CpG DMR spanning approximately 1.7 kb, supporting interpretation of the cluster as a coordinated regional methylation signal. Additional same-tissue evidence was observed in the GSE60655 endurance-training cohort, where all five testable TNXB CpGs changed in the same direction as in the discovery cohort, including two nominally significant sites. Taken together, the locus-level, region-level, and cross-cohort findings identify TNXB as a priority region for further investigation of exercise-associated skeletal-muscle methylation.

TNXB encodes tenascin-X, an extracellular-matrix glycoprotein involved in tissue structural organization. Extracellular-matrix remodeling is an important component of skeletal-muscle adaptation to repeated mechanical loading, contributing to force transmission, tissue repair, and structural remodeling [31,32,33]. The coordinated hypomethylation observed across the TNXB region therefore provides a biologically plausible link between exercise-associated epigenetic remodeling and extracellular-matrix adaptation. This finding generates a testable hypothesis that changes in methylation at the TNXB region may accompany transcriptional or structural remodeling during training. Matched transcriptomic and functional measurements will be important for determining how these methylation changes relate to TNXB expression and extracellular-matrix function.

The genomic context of TNXB adds an important layer to this interpretation. The TNXB cluster lies within the extended MHC/RCCX region, which is characterized by substantial genetic polymorphism, mQTL effects, and copy-number variation. Because matched genotype, ancestry, and CNV information is unavailable in the public cohorts analyzed here, future genotype-aware studies will be important for distinguishing exercise-associated methylation changes from underlying genetic or population-structure effects. Chromatin-state annotations from Roadmaps E107 and E108 also indicate differences in the regulatory context of this region across skeletal-muscle reference epigenomes. Integrating genotype-aware analyses with matched RNA-seq, ATAC-seq, and functional measurements may therefore help determine how TNXB-region methylation relates to transcriptional regulation and extracellular-matrix remodeling during training.

### 3.3. SIM1- and PIWIL4-Related Candidate Loci

cg06639166 was the locus with the largest effect size in the 21-CpG shortlist, located at chr6:100,913,923 near SIM1, and is the only CpG that passed the strict L5 threshold. Given SIM1’s role in energy intake and body-weight regulation, cg06639166/SIM1 represents a candidate lead linking exercise response to whole-body energy metabolism. However, several factors suggest caution. SIM1 is lowly expressed in GTEx vastus lateralis, cg06639166 is annotated as a polycomb-repressed state in Roadmap E108, and in GSE60655, it exhibited only directional concordance without reaching significance. Therefore, this locus should be interpreted as a possible distal-regulatory or systemic-metabolism-related methylation lead, rather than as direct evidence of local muscle SIM1 transcriptional change.

Located in the PIWIL4 TSS1500 region, cg00075402 has not been reported previously in the EWAS Catalog or PubMed. It is also not a member of any published epigenetic clock. This locus is hypomethylated in HIIT skeletal muscle. In contrast, this locus showed no association in the paired analysis of GSE213363 (Table S7); the nominal, direction-reversed screening association did not survive correction. This result should not be interpreted as reproduction or confirmation; instead, it suggests that the PIWIL4-related methylation signal may depend on tissue, disease context, and training modality. cg00075402 was not testable in GSE60655 because it is absent from the 450 K array.

Both the SIM1- and PIWIL4-related loci should therefore be treated as hypothesis-generating signals. While they illustrate the pipeline’s ability to capture diverse biological leads beyond the single TNXB region, their mechanistic interpretation still requires support from matched transcriptomics, small RNA-seq, and functional follow-up of the target regions.

### 3.4. Interpretive Boundaries of the DNAm Age Clock

The paired within-participant analysis is the primary evidence; among 15 participants, only Horvath2013 remained significant after correction (Δ = +1.40 years), with Horvath2018 SkinBlood and PhenoAge being nominal only. The whole-cohort comparison, in which the two groups largely comprised the same participants, is descriptive and showed the same direction. These findings are best interpreted as a tissue-specific, training-associated methylation change captured by a single chronological clock in a small paired sample, rather than evidence that HIIT alters systemic biological aging. The pace-of-aging measure showed no change, consistent with this interpretation, although incomplete probe coverage limited all five clocks when applied to these data (Section 4.11).

### 3.5. Cross-Cohort Robustness

The three external datasets provided complementary cross-cohort evaluations under different experimental and biological conditions. GSE268211 provided a partner-laboratory, same-platform, same-tissue evaluation, with transfer AUROCs of 0.96–1.00 across the five fitted models (Table S6). Given its small size (10 samples from five paired participants), these estimates were accompanied by unstable confidence intervals and variable classification accuracy. Accordingly, this cohort provided a preliminary consistency check for model transfer under closely matched technical and tissue conditions.

GSE213363 provided a more challenging cross-tissue, cross-disease, and cross-training-modality evaluation. The pipeline achieved a mean AUROC of 0.673 ± 0.065, indicating modest generalization in this substantially different context. At the locus level, one of the 21 candidates showed an FDR-significant, direction-concordant change, whereas the remaining candidates did not show comparable evidence of transfer. These results suggest that part of the candidate signal may persist across substantially different biological contexts, although its overall reproducibility was attenuated relative to the same-tissue evaluations.

GSE60655 provided a same-tissue evaluation across a different array generation and training modality. All 15 testable candidates, including all five TNXB CpGs, changed in the same direction as in the discovery cohort, although none remained significant after BH-FDR correction. These findings provide evidence of directional consistency in skeletal muscle across distinct exercise paradigms, with the TNXB cluster contributing a coherent regional component. Given the cohort-wide directional background, the results from this dataset are most informative at the panel and regional levels, while evidence for individual-locus replication remains limited.

### 3.6. Relationship to Existing Studies of Exercise Epigenetic Markers

This study extends existing exercise epigenetic research by introducing a structured framework for candidate generation and evaluation in longitudinal datasets with repeated sampling and complex sub-cohort structures. By integrating batch-aware screening, subject-grouped nested cross-validation, three-axis consensus screening, and cross-cohort evaluation, the framework addresses several analytical challenges that commonly arise in small longitudinal exercise cohorts, including repeated measurements from the same participants, uneven representation across time points, and structured sub-cohort effects.

At the candidate-locus level, the 21-CpG shortlist was derived from consistency across binary, multi-class, and temporal tasks rather than from a single differential-methylation contrast. In the K-curve comparison, the three approaches showed similar performance at K = 21, while the present pipeline performed competitively across the K = 100–500 range. The principal value of the framework therefore lies in integrating cross-task consistency, participant-aware model evaluation, and cross-cohort follow-up within a single candidate-prioritization workflow. This provides a complementary methodological perspective to conventional differential-methylation analyses and may be particularly useful for prioritizing a focused set of candidates in small, longitudinal, high-dimensional methylation datasets.

### 3.7. Limitations

This study’s limitations include sample size, sex distribution, and sub-cohort structure. Specifically, the discovery cohort consisted of 195 samples from 52 independent individuals, with a male-biased sex distribution and certain time points exclusively present in the Inter2 sub-cohort. To mitigate the influence of this structure on the main classification results, we employed stratified screening within study group, individual_id-grouped cross-validation, male-only analysis, and sex-covariate analysis. Both the male-only and sex-covariate results closely matched the baseline AUC. These findings suggest that the main classification signal is not driven by within-cohort sex composition; however, long-duration time points remained linked to the Inter2 sub-cohort, and effect estimates in the smallest (Main) sub-cohort were systematically larger than in the other strata. Therefore, sex-specific biology and long-term time-point effects still require validation in more balanced independent cohorts.

While the cross-cohort evaluation encompassed diverse laboratories, tissues, platforms, and disease contexts, these differences inherently limit the extrapolation of the conclusions. For instance, GSE268211 included only 10 samples (five paired subjects), leading to limited confidence-interval stability. GSE213363 differed substantially from the discovery cohort in tissue and disease context, in which all 21 shortlist sites were testable and only one CpG passed correction. Similarly, GSE60655, an endurance cohort, yielded 0/15 FDR-significant CpGs and exhibited limited 450 K/EPIC probe overlap. In both external training cohorts, time point was aligned with array position on the BeadChip, so that technical and training effects could not be fully separated in this regard; external comparisons were therefore restricted to direction and bounded performance checks. Both the discovery and public external cohorts provided methylation data without matched genotype, ancestry, or CNV information. Consequently, mQTL, population-structure, copy-number, or SNP-affected-probe effects cannot be excluded, particularly for extended-MHC signals like TNXB. The current results support transferability at the same-tissue directional level, but they do not prove that the 21-CpG shortlist performs identically across all exercise or disease contexts. Subsequent work will require larger-scale, same-tissue, independent-laboratory evaluation cohorts with more closely matched sampling designs and genotype-aware annotation.

The 21-CpG shortlist remains an exploratory candidate set. The screening thresholds remain heuristic; locus-level inference in this study rests on the participant-aware mixed models rather than on the screen itself. Although the TNXB cluster, cg06639166/SIM1, and cg00075402/PIWIL4 each exhibited statistical and cross-cohort evidence at various levels, this study lacked matched RNA-seq, small RNA-seq, ATAC-seq, genotype/ancestry information, CNV data, or functional perturbation experiments for the same samples. Bulk vastus lateralis biopsies contain mixed cell populations, and methylation differences may partly reflect shifts in fiber-type or cellular composition rather than within-cell regulation; cell-type deconvolution references for skeletal muscle remain limited [34]. Therefore, these loci should not be interpreted as candidate markers with completed mechanistic confirmation. Subsequent studies should prioritize the simultaneous measurement of methylation, expression, genotype/ancestry, copy number, and small RNA in matched biopsies, along with functional follow-up of the key regions.

The DNAm clock results also present clear interpretive boundaries. The descriptive whole-cohort pattern, coupled with the DunedinPACE null finding, is most consistent with a tissue-specific, training-associated response. However, the within-subject longitudinal analysis was limited to 15 paired Inter2 subjects, and only Horvath2013 remained significant after BH-FDR correction. Probe coverage further limited all five clocks on these data, with 35–60% of model CpGs present on the preprocessed array (Table S9). Consequently, these results cannot infer that HIIT alters the systemic biological rate of aging. Long-term training, detraining, and multi-tissue sampling will help distinguish the relationships among transient tissue remodeling, persistent epigenetic imprint, and a systemic aging proxy.

### 3.8. Significance and Future Work

The significance of this study lies in establishing a structured candidate-prioritization framework for longitudinal exercise methylation data. The framework integrates within-sub-cohort statistical estimation, subject-grouped cross-validation, multi-task consensus screening, and cross-cohort evaluation, thereby linking candidate selection with participant-aware model assessment and external follow-up. These design principles may provide a useful analytical reference for other high-dimensional longitudinal methylation studies characterized by limited sample sizes, repeated measurements, multiple time points, and complex metadata structures.

The 21-CpG shortlist also highlights several biologically relevant directions for subsequent investigation. The TNXB regional signal motivates further examination of extracellular-matrix remodeling in exercise-adapted skeletal muscle, while cg06639166 near SIM1 raises questions regarding metabolic and distal-regulatory processes. The PIWIL4-associated locus provides an additional lead for investigating potential links with small-RNA-related regulation. These signals therefore define a set of testable molecular hypotheses that can be examined through complementary transcriptomic, genetic, epigenomic, and functional approaches.

Future work should prioritize four directions: matched multi-omics profiling, longer-term training and detraining cohorts, functional investigation of key regions, and larger independent cohorts with more balanced participant characteristics. Integrating methylation data with matched RNA-seq, small RNA-seq, genotype and ancestry information, copy-number data, and chromatin-accessibility measurements from the same biopsy material would help determine how the prioritized CpGs relate to transcriptional and regulatory changes. Repeated sampling during longer-term training and detraining could further distinguish transient training-associated remodeling from persistent epigenetic changes. Functional studies of the TNXB region, the locus near SIM1, and the PIWIL4 TSS1500 region could then assess the regulatory consequences associated with these methylation signals. Finally, replication in larger skeletal-muscle cohorts with more balanced sex distributions and closely matched sampling designs will be important for establishing the reproducibility and broader relevance of the candidate set. These studies may help advance the current pipeline-derived candidates from exploratory methylation signals toward mechanistic interpretation and evaluation of their broader biological relevance.

## 4. Materials and Methods

### 4.1. Cohorts and Samples

This study used skeletal muscle biopsy samples from the vastus lateralis collected during a 12-week high-intensity interval training (HIIT) intervention trial, measuring DNA methylation profiles with the Illumina Infinium MethylationEPIC v1.0 BeadChip. The cohort comprised 195 samples (149 from male and 46 from female participants) contributed by 52 unique participants (32 male, 20 female). Based on recruitment and intervention schedules, subjects were assigned to 3 sub-cohorts (Main, Inter1, and Inter2).

Each subject provided biopsies at one or more of the following time points: baseline, 4-week HIIT, 8-week HIIT, and 12-week HIIT, along with non-intervention control samples. Based on this sampling design, we defined three classes of downstream modeling task labels: (i) a binary task (HIIT vs. control), where the HIIT class comprised the 4-week, 8-week, and 12-week HIIT samples (n = 102) and the control class comprised the baseline and non-intervention control samples (n = 93); (ii) a multi-class 5-way task distinguishing non-intervention control, baseline, 4W, 8W, and 12W, with sample counts of 25, 68, 70, 17, and 15, respectively; and (iii) a temporal four-class task including only the HIIT intervention time series (0W = 68, 4W = 70, 8W = 17, 12W = 15) to characterize changes along the ordinal axis of training duration, where the 8W and 12W time points occurred only in the Inter2 sub-cohort. Baseline and non-intervention control samples were assigned to a common control class because neither represented a training-exposed state at the time of biopsy; the two control types were retained as separate levels on the multi-class axis, where their distinction was tested directly. These values represent sample counts; the discovery cohort included 52 unique participants, with the unique-participant counts at the study group × timepoint level detailed in Table 3. The participant-level sampling structure, including the participants who contributed to two sub-cohorts and the time points available for each, is summarized in Figure S4. Metadata were stratified by study group and time point, and all three task labels were linked to the same sample-to-subject mapping table. All subsequent statistical screening and modeling shared this single mapping, ensuring consistent label definitions across tasks. To prevent repeated samples from the same participant from being treated as independent subjects or split across training and test folds, we used individual_id as the independent participant identifier across all downstream resampling, nested cross-validation, and bootstrap analyses. The participant was therefore the independent unit of analysis throughout, whereas the sample was the unit of observation.

The table shows sample counts, sex distribution, and unique-participant numbers per study group x time point in GSE171140 (discovery dataset; n = 195 samples from 52 unique participants). Sub-cohort unique-participant counts sum to 69 because 17 participants contributed to two sub-cohorts. Both baseline and non-exercise control samples were assigned to the control class in the binary HIIT versus control classification task. In selected cells, the number of samples exceeds the number of unique participants because a single participant contributed two arrays from the same biopsy time point (technical replicates); these were retained as separate observations sharing the participant grouping; no separate biopsy-level random effect was fitted. In sensitivity analyses of the binary-status mixed model, averaging the β values of each of the three technical-replicate pairs or retaining only the first array in each pair reduced the data to 192 observations. For the M-scale analysis, pairwise averaging was performed on the β scale before transformation to M values. Under both alternatives, the same 20 of 21 candidate CpGs remained significant at BH-FDR < 0.05 across the candidate set on both the β and M scales.

### 4.2. Methylation Array Preprocessing

Methylation data for the discovery cohort were obtained as the processed β-value series matrix deposited with GSE171140; no raw intensity files were reprocessed for this cohort. As described by the depositors, β values were derived from the methylated and unmethylated intensities and adjusted for the Infinium type I and type II probe designs by β-mixture quantile normalization, after which the values were converted to the M scale and corrected for plate and plate position. Probes were removed by the depositors when they carried missing β values, a detection *p* value above 0.01, or a bead count below three in at least 5% of samples, and when they were non-CpG probes, probes aligning to multiple genomic locations, probes on the sex chromosomes, or probes overlapping the published European-ancestry SNP-associated probe list; 763,222 of the 865,859 positions of the EPIC v1.0 design remained. From this matrix, we further removed 121,507 probes with more than 10% missing β values across the 195 samples and 273,119 probes with a probe-wise standard deviation below 0.02, leaving a 195 × 368,596 β-value matrix. Missingness in this cohort was structured by sub-cohort rather than distributed at random, so the first of these two filters amounted to a complete-case intersection across the three sub-cohorts and the resulting matrix contained no missing values.

Next, we applied variance-based top-k pre-filtering to this matrix. Specifically, we calculated the probe-wise β-value variance across all 368,596 probes, ranked the probes in descending order of variance, and retained the top 95,477 CpGs for downstream analysis. This top-k threshold served as a variance-ranked pre-filter to reduce the dimensionality of the downstream modeling problem, resulting in a final output matrix of 195 × 95,477. Because this ranking was computed on all 195 samples, its effect on model evaluation was quantified separately by the fold-contained sensitivity analysis described in Section 4.7.5.

Sample-level metadata, including the study group (Main/Inter1/Inter2), sex, time point, individual_id, and task-label fields, were matched one-to-one with the β-value matrix above by sample ID. At this stage, the study group labels were not included in any β-value transformation or matrix-level adjustment; instead, they were retained solely as metadata to serve as a stratification factor in the downstream batch-aware statistical screening (see Section 4.3). No transformation other than the probe filtering described above and the variance-ranked pre-filter was applied to the β-value matrix at this stage, and all 195 samples entered downstream modeling; no sample was removed for quality reasons, and after probe filtering, no sample carried a missing value.

To improve processing efficiency and facilitate subsequent batch-aware testing and subject-grouped cross-validation calls, we consolidated this 195 × 95,477 matrix together with the corresponding metadata table (containing the study group, time point, and individual_id columns) into a unified input interface for the pipeline. All downstream analysis modules read directly from this interface, performing no secondary preprocessing at intermediate stages. To characterize the main sources of variation in the discovery feature space, we analyzed the variance structure of the 195 × 95,477 β-value matrix using a descriptive PERMANOVA-style approach with Euclidean distance and 999 permutations. The resulting variance components, reported in Supplementary Table S3, were used to document the relative contributions of subject identity, study group/sub-cohort, sex, and training-time labels before downstream modeling.

### 4.3. Batch-Aware Statistical Screening

We performed batch-aware statistical screening in parallel for the three task classes using the 195 × 95,477 β-value matrix. Locus-level evidence was first estimated within each study-group stratum and subsequently combined into a single CpG-level result. For each CpG, task axis, stratum, and cross-validation (CV) fold, the within-group test yielded a *p* value and a task-specific effect summary. Valid stratum-specific *p* values were then combined using Fisher’s method [35], and the corresponding effect summaries were arithmetically averaged:

(1) $$S_{i,a,f}=-2\;\sum_{g\in G_{i,a,f}^{valid}}\ln\left(p_{i,a,g,f}\right)$$

(2) $$p_{i,a,f}^{combined}=P\left(\chi_{2k_{i,a,f}}^{2}\geq S_{i,a,f}\right),\;k_{i,a,f}=\left|G_{i,a,f}^{valid}\right|$$

(3) $${\bar{e}}_{i,a,f}=\left(\frac{1}{k_{i,a,f}}\right)\sum_{g\in G_{i,a,f}^{valid}}e_{i,a,g,f}$$

Consequently, each CpG on each task axis and CV fold yielded a Fisher-combined *p* value and an averaged task-specific effect summary for the L5/L6 threshold decision. Stratum-specific tests contributed to these results only when sample-size and factor-level requirements were met; invalid strata were omitted rather than imputed. A stratum contributed to the binary axis only when at least three samples were available in each of the two classes, to the multi-class axis only when at least two classes were present and each present class contained at least three samples, and to the temporal axis only when the stratum contained at least five samples spanning at least two distinct time points. When no stratum met these requirements for a given axis, the test for that axis was carried out once on the pooled cohort instead of being combined across strata.

Within this framework, we defined three task classes. First, the binary task compared HIIT with the control using a Welch two-sample *t*-test in strata containing sufficient samples in both groups. This task used Cohen’s d as the effect size and returned a Fisher-combined *p* value and an averaged signed Cohen’s d [36]. Second, the multi-class task distinguished among control, baseline, 4W, 8W, and 12W using a one-way ANOVA across the classes that were present and sufficiently represented in each stratum. This analysis used the F statistic as the effect size, returning a Fisher-combined *p* value and an averaged F statistic. Third, the temporal task modeled the HIIT intervention time levels (0W, 4W, 8W, and 12W) using OLS regression within each stratum, requiring at least two valid time levels. The Main and Inter1 cohorts contributed shorter-duration levels, whereas the 8W and 12W levels occurred only in Inter2. We neither imputed missing time levels nor treated them as present. In this regression, the ordinal time-point code served as the independent variable and the β value as the dependent variable, with R^2^ and slope summarizing the effects. The final outputs were the Fisher-combined *p* value, averaged R^2^, and averaged slope.

We established two screening thresholds: Level 5 (L5) and Level 6 (L6). L5 required a Fisher-combined *p* value < 0.05 combined with an absolute averaged Cohen’s d > 0.30, an averaged F > 4.0, and an averaged R^2^ > 0.20. Similarly, L6 required a Fisher-combined *p* value < 0.05 combined with an absolute averaged Cohen’s d > 0.20, an averaged F > 3.0, and an averaged R^2^ > 0.15. Both levels required satisfying the *p*-value and effect-size thresholds simultaneously. Consequently, CpGs meeting only one criterion were excluded from the corresponding task-axis shortlist.

We applied the L5/L6 thresholds at two levels: nested cross-validation and a single full-cohort screening. For the nested cross-validation, we re-executed the batch-aware screening described in Section 4.3 within each outer training fold to obtain the training feature subset for that fold. Under the L6 threshold, each fold passed an average of 33,586 ± 6739 binary, 2075 ± 906 multi-class, and 299 temporal CpGs, which defined the input scale for feature screening within CV. For the single full-cohort screening, the same L6 threshold passed 12,978 binary, 705 multi-class, and 30 temporal CpGs. These full-cohort task-axis sets were then used for the three-axis intersection described in Section 2.6, the funnel and Venn diagrams in Figure 1B,C, and the 21-CpG shortlist reported in Results Section 2.3. Results Section 2.3 and Table 1 detail the L5 and L6 candidate shortlists along with the CpG counts at each level.

To assess robustness to threshold selection, we repeated the complete workflow comprising per-batch testing, Fisher’s-method combination, and three-task intersection, across 10 candidate levels (Level 1 to Level 10) corresponding to different Fisher-combined *p*-value and effect-size thresholds. This sensitivity sweep confirmed monotonic changes in the candidate set as thresholds were tightened or relaxed. Classification performance formed a plateau over the neighboring levels and did not improve when the thresholds were relaxed further, and L5 and L6 were retained on that basis as the stringent and the main-analysis levels, respectively. Here, we report the stricter L5 and the L6 main-shortlist level, with the corresponding per-axis pass counts and three-axis intersection counts shown in Figure 1B,C and Results Section 2.3.

Because the 195 samples derive from 52 participants, locus-level inference for the candidate CpGs was carried out with linear mixed-effects models (statsmodels version 0.14.6) rather than with the screening statistics described above [19,37]. We modeled each CpG with a random intercept for participant, with training status (or, in the longitudinal specification, time point relative to baseline) together with sub-cohort and sex as fixed effects, and the models were fitted by restricted maximum likelihood on both the β scale and the M scale (M = log2(β/(1 − β))). The same specification was applied to all 368,596 preprocessed CpGs so that the candidates could be placed on a genome-wide scale, and *p* values were corrected by the Benjamini–Hochberg procedure both within the candidate set and across the genome. Two further participant-aware models address the sub-cohort structure: a longitudinal model restricted to Inter2, the only sub-cohort in which all four time points were available, and a common baseline-to-4-week model fitted across the three sub-cohorts with a training-by-sub-cohort interaction term.

### 4.4. Subject-Grouped Nested Cross-Validation

For downstream modeling, we implemented nested cross-validation using scikit-learn’s (version 1.6.1) GroupKFold, with individual_id serving as the grouping key (groups) [38]. The outer split comprised 5 folds to estimate the generalization AUC. Within each outer training fold, the inner split performed a 3-fold GridSearchCV for hyperparameter search, such as tuning the regularization strength C for LR and max_depth for RandomForest [39]. Assigning the outer folds by individual_id ensured that all samples from a given subject remained within the same fold. Furthermore, the binary, multi-class, and temporal tasks shared the identical fold_splits, enabling direct comparison of per-fold results across tasks.

Within each outer training fold, we constructed the complete pipeline by serially chaining a batch-aware feature selector, a StandardScaler, and a final classifier. We fitted this pipeline on the training fold in the sequence selector → scaler → classifier and subsequently executed transform and predict on the corresponding test fold. Fitting the selector enabled the batch_aware = True parameter, which used a lookup mechanism to retrieve the study group labels for the sample subset in that fold. The selector then re-executed the per-batch testing and Fisher’s-method combination described in Section 4.3 within the training fold to output the CpG subset for that fold. Next, the StandardScaler estimated the mean and standard deviation of each retained CpG on the training-fold subset and used these parameters to transform both the training and test folds. Finally, the classifier was fit on the standardized training matrix to output predicted probabilities and labels on the test matrix.

To account for unequal sample sizes across classes, we trained the classifiers with class_weight = ‘balanced’. We evaluated three classifier families across all three tasks: LR-L1 (Lasso), LR-L2 (Ridge), and RandomForest. Hyperparameters were optimized using an inner GridSearchCV with ROC AUC as the scoring metric. The search grids included C ∈ {0.01, 0.1, 1, 10} for LR, and max_depth ∈ {3, 5, 8, None} with n_estimators = 500 for RandomForest. For the binary task, we also evaluated the Saga solver and an extended grid of 0.001–1000 as a sensitivity reference.

For each outer fold, we saved the predicted probabilities, true labels, AUC, accuracy, the list of CpGs selected by the selector, and the hyperparameters chosen by GridSearchCV in per_fold_metrics.csv and selected_cpgs_long.csv. Additionally, we saved the JSON table of the same fold split (fold_splits.json) and the selector-hit CpG list for each fold. These files were preserved for the downstream model-comparison, subject-level bootstrap, and permutation analyses (Section 4.7.3) and for cross-task comparisons.

### 4.5. Multi-Model Evaluation

In this part of the analysis, we extended the 3-classifier (LR-L1/LR-L2/RandomForest) baseline framework described in Section 4.4 and Section 4.5 model families by adding ElasticNet and LightGBM (version 4.6.0). For both LR and ElasticNet, we uniformly switched to the Saga solver and an expanded regularization-strength grid. To ensure a fair comparison, all 5 models shared the fold_splits and selector output of Section 4.4. This design was chosen because a single algorithm is prone to algorithm-dependent overfitting on small-sample, high-dimensional data, whereas a parallel comparison of multiple model families can reveal whether the candidate signal depends on a particular classifier structure.

We configured the hyperparameter grids for each model as follows: LR-L1 and LR-L2 used the Saga solver for high-dimensional penalized regression, with a C grid of {0.001, 0.01, 0.1, 1, 10, 100, 1000} and class_weight = ‘balanced’ enabled; ElasticNet used l1_ratio = 0.9 with the same C grid as LR; RandomForest used n_estimators = 500, max_features = ‘sqrt’, and max_depth ∈ {3, 5, 8, None}; and LightGBM [40] used num_leaves = 15, learning_rate = 0.05, and n_estimators = 500. Within each outer fold, a 3-fold GridSearchCV selected the optimal C or max_depth, using ROC AUC as the uniform inner scoring metric. LightGBM was therefore fitted with these fixed settings, no LightGBM hyperparameter having been entered into the inner search.

Within each outer fold, each model output the outer test AUC and a list of the top-100 most important features. Feature importance was determined by the absolute value of coefficients for LR and ElasticNet, Gini importance for RandomForest, and gain for LightGBM. Because these importance measures are computed on different scales, they were interpreted within, rather than across, model families. To measure cross-model feature overlap, we computed the pairwise Jaccard coefficient of these top-100 lists among the 5 models. We discuss the interpretation of this Jaccard measure in relation to the characteristics of the signal distribution in Discussion Section 3.1.

### 4.6. Three-Axis Intersection Candidate Screening

Building on the batch-aware screening output from Section 4.3, we defined 3 axes: (a) a binary HIIT vs. control axis based on Cohen’s d and a Fisher-combined *p* value; (b) a multi-class 5-way axis based on the ANOVA F statistic and a Fisher-combined *p* value; and (c) a temporal four-class ordinal axis based on OLS slope, R^2^, and a Fisher-combined *p* value. All three axes shared the same batch-aware Fisher’s-method combination framework, which defined significance using consistent per-study-group testing and sub-cohort combination rules. These 3 parallel axes established the foundation for the downstream consensus shortlist design.

Each axis was assigned two sets of thresholds: L5 (more stringent) and L6 (the main-analysis threshold chosen after a sensitivity sweep). The binary axis required an absolute Cohen’s d > 0.30 (L5) or > 0.20 (L6). The multi-class axis required F > 4.0 (L5) or > 3.0 (L6), and the temporal axis required R^2^ > 0.20 (L5) or > 0.15 (L6). Crucially, all axes simultaneously required a Fisher-combined *p* value < 0.05. This three-axis intersection design aimed to create a cross-task-consistent, conservative prioritization shortlist. For inclusion in the final list, each candidate CpG had to meet the binary, multi-class, and temporal criteria simultaneously. This workflow yielded two candidate shortlists, L6 and L5, from the study cohorts. Their specific counts, biological annotation, set shrinkage under synthetic injection, and coordinated TPR/injection-defined FDR are presented in Results Section 2.1 and Section 2.3, and in Table 1.

### 4.7. Evaluation Suite

To assess the robustness of the screening pipeline across multiple dimensions, we developed a suite of four evaluation protocols: synthetic injection, reproducibility, statistical robustness, and methodological comparison. This section details the design of these protocols; their corresponding numerical results are presented in Results Section 2.1 and Section 2.2, respectively.

#### 4.7.1. Synthetic Injection Benchmark

We first established a synthetic injection benchmark. This involved shuffling the labels of the original β matrix across samples to create a null-permuted background. Onto this background, a known number of true-positive CpGs were injected. Scenarios were independently constructed using 3 random seeds and two injection modes: (a) single-axis, where the signal was injected only on the binary axis (d ∈ {0.3, 0.5, 0.7, 1.0}); and (b) coordinated 3-axis, where the same CpG received the corresponding signal simultaneously on all three axes (binary, multi-class, and temporal, d ∈ {0.2, 0.3, 0.5, 0.8}). Non-injected CpGs served as negative controls. The evaluation protocol entailed performing the complete batch-aware screening on the post-injection matrix, comparing the resulting shortlist with the ground truth (injection positions), and computing the TPR, FPR, and set shrinkage ratio. Specific values are provided in Results Section 2.1. Injected effects were introduced by shifting the β values of the target group by d times the within-group standard deviation of each selected CpG. Five hundred permutations bound the empirical *p* value at 0.002 and kept the full-pipeline rerun computationally tractable.

#### 4.7.2. Reproducibility (Seed Stability)

To assess the pipeline’s determinism, we ran the entire pipeline independently using multiple random seeds. We then compared the pairwise Jaccard coefficients of the candidate CpG lists generated by each seed as a measure of reproducibility. The specific values are provided in Results Section 2.1.

#### 4.7.3. Statistical Robustness

For the final model, we implemented a three-tier statistical robustness protocol:

(a) Subject-level bootstrap: We performed 1000 resamples of the 52 independent subjects. The resampling unit was the subject, not the sample, to preserve the within-subject multi-timepoint structure. Each bootstrap re-evaluated the outer test AUC using the resampled subject subset, yielding the 95% confidence interval of the baseline binary model AUC. Intervals were taken as the 2.5th and 97.5th percentiles of the bootstrap distribution.

(b) Permutation null: We shuffled only the case-control labels 500 times, preserving the subject pairing structure (a within-individual shuffle). Each shuffle involved rerunning the complete nested CV and comparing the observed AUC with the null distribution to obtain a one-sided *p* value. Labels were exchanged only among samples of the same participant, so that the composition of the cohort and the number of samples per participant were held fixed under the null.

(c) Pooled out-of-fold model comparison: Because the five outer-fold models shared most of their training data, the model families were compared on pooled out-of-fold predictions rather than fold by fold. The out-of-fold predicted probabilities of the five folds were pooled, and participants were resampled 2000 times to obtain a confidence interval for each pooled AUROC and for each paired between-model difference, with two-sided *p* values taken from the proportion of resamples in which the paired difference reversed the sign. This resampling was enacted on the stored out-of-fold predictions and therefore represents sampling variation among participants, not the variability of model training and feature selection.

Specific confidence intervals and *p* values are presented in Results Section 2.2 and Discussion Section 3.1.

#### 4.7.4. Method-Comparison K-Curve

To assess the predictive performance of our batch-aware three-axis intersection pipeline relative to classical differential methylation analysis, we designed a method-comparison K-curve protocol. This protocol involved a three-way parallel evaluation of limma (https://bioconductor.org/packages/limma/, accessed on 21 September 2026) (moderated t based on M values) [41], minfi (https://bioconductor.org/packages/minfi/, accessed on 21 September 2026) (per-CpG *t*-test) [42], and our pipeline for a binary classification task. Within each outer fold, we recomputed the three rankings using only that fold’s training subjects. We then used the top-K CpGs from each ranking (K ∈ {21, 50, 100, 500, 1000}) to train an LR-L2 classifier. The AUC was subsequently computed on the corresponding test subjects, yielding an AUC table of 5 K × 3 method × 5 fold. This approach enabled a direct comparison of the predictive ability of the three rankings under a unified subject-grouped CV framework and with an equal number of features. The specific AUC table and inter-method differences are presented in Results Section 2.1, Table S5, and Figure 2D.

#### 4.7.5. Fold-Contained Variance Filtering Sensitivity Analysis

The variance-ranked pre-filter of Section 4.2 was computed on all 195 samples before the outer folds were formed. To quantify the effect of this ordering, the complete nested cross-validation was repeated with the variance ranking recomputed within each outer training fold and applied unchanged to the corresponding test fold, all other settings being identical, and the five model families were refitted under this configuration. The resulting estimates are reported in Results Section 2.2.

### 4.8. External and Cross-Cohort Evaluation

To evaluate the cross-cohort generalizability of the candidate shortlist and the pipeline, we established transfer and retraining protocols across three independent public datasets. These datasets represented three distinct scenarios: partner-laboratory same-platform, cross-disease same-platform, and cross-array same-tissue.

#### 4.8.1. Partner-Laboratory Transfer (GSE268211)

We used GSE268211 (a partner laboratory, EPIC v1.0, skeletal muscle biopsies, 5 paired baseline and 8-week training samples, n = 10) for the partner-laboratory transfer protocol. Under this protocol, the feature-screening step (batch-aware funnel) and the final 5 models were fitted on the complete GSE171140 cohort as the training dataset. We then applied the fully fitted pipeline directly to GSE268211 for prediction. Because the training and external test sets originated from two fully independent GEO resources, the transfer process was naturally partitioned at the dataset level. This protocol yielded the external transfer AUC and accuracy for each of the 5 models with bootstrap confidence intervals; these values are detailed in Results Section 2.5, Table 2 and Table S6.

#### 4.8.2. Cross-Disease Retraining (GSE213363)

We used GSE213363 (Jenkins lab, EPIC v1.0, whole blood, resistance and aerobic training in a population of women with polycystic ovary syndrome, 56 paired pre-vs-post, n = 112) for the cross-disease + cross-tissue evaluation. Within GSE213363, we retrained the pipeline using 5-fold subject-grouped cross-validation. Within each outer fold, top-95 K variance pre-filtering, the batch-aware Welch + Fisher’s-method combination, L6 threshold screening, and LR-L2 fitting were re-executed using only the training fold, after which the AUC was estimated on the test fold. This design simultaneously evaluated pipeline transferability across different tissues and clinical contexts, with the specific per-fold AUC values provided in Results Section 2.5. In addition, all 21 candidate CpGs were tested directly in GSE213363 using exact paired Wilcoxon signed-rank tests across the 56 pre-post pairs, with Benjamini–Hochberg correction across the 21 candidates and participant-level bootstrap confidence intervals for the paired change in β (Table S7).

#### 4.8.3. Cross-Array, Same-Tissue Evaluation (GSE60655)

For the cross-array + cross-modality same-tissue evaluation, we used GSE60655 (Nitert/Lindholm, Illumina 450 K, vastus lateralis, 3-month aerobic endurance training, 34 analytical samples from 17 paired participants (baseline and post-training)). The candidate shortlist from the pipeline was directly subjected to an exact two-sided Wilcoxon signed-rank test on GSE60655 to determine the direction sign and raw *p* value of each candidate CpG, with the paired *t*-test retained as a sensitivity analysis; concurrently, we performed independent subject-grouped CV on GSE60655 to estimate the AUC. Because certain candidate CpGs were exclusive to the EPIC platform, we restricted the cross-array direction concordance and significance comparisons to the overlapping probe subset of the two arrays. This protocol simultaneously evaluated the direction concordance and raw significance of the candidates under array-generation differences and alternative training modalities, as detailed in Results Section 2.5 and Figure 4C and Figure 5. Because the shortlist was predominantly hypomethylated and contained correlated TNXB probes, direction concordance was additionally assessed against a matched background of probes drawn from the same cohort, and a sign-flip test was applied within participants to the panel as a whole; both analyses are reported in Results (Section 2.5).

### 4.9. Enhanced Annotation

We integrated three classes of locus-level annotations for each candidate CpG. The first class, derived from the Illumina EPIC v1.0 manifest, provided the chromosomal coordinates of the CpG, the nearest gene, the UCSC RefGene group (TSS1500/TSS200/5′UTR/Body/3′UTR, etc.), and the island context [12]. The second class, obtained by querying the EWAS Catalog (NHGRI-EBI Catalog), retrieved the number of associations and the top previously reported trait for each candidate CpG to assess prior knowledge [43]. The third class, based on the annotatr R package (https://bioconductor.org/packages/annotatr/, accessed on 21 September 2026), annotated each candidate on hg19 to CpG island/shore/shelf/open sea and to gene structural regions such as gene body/promoter/5′UTR/3′UTR/intergenic [44]. Probe-level annotation used the EPIC v1.0 B4 manifest as distributed in the IlluminaHumanMethylationEPICanno.ilm10b4.hg19 annotation package version 0.6.0) (hg19); the EWAS Catalog was queried through its public application programming interface on 25 May 2026; and annotatr was run against its hg19 built-in annotation sets. Exact package versions are recorded in the environment file of the analysis repository.

For chromatin state annotation, we used the Roadmap Epigenomics 15-state ChromHMM model to query each candidate locus across two skeletal muscle reference epigenomes: E108 (female skeletal muscle), E107 (male skeletal muscle). Each locus was assigned to 1 of 15 states (such as active enhancer, bivalent, polycomb-repressed, or quiescent), which, together with the FANTOM-linked regulatory context, established the biological background [20,45]. We integrated gene expression data using the median TPM from GTEx v8 in vastus lateralis tissue, matched to the candidate-associated genes [46].

We integrated the three classes of annotation mentioned above into a single candidate-CpG annotation table (Table 1 in the main text). Each row of Table 1 represents a candidate CpG, detailing its ID, chromosomal coordinates, associated gene, region class, island context, and the three-axis effect sizes (Cohen’s d/ANOVA F/temporal R2). Additional fields include the E108 (female skeletal muscle) chromatin state, EWAS prior hit count, and the direction sign and raw *p* value (Wilcoxon paired) from the same-tissue cross-array directional-concordance check in GSE60655. E107 (male skeletal muscle) and E120 (HSMM cell line) were also queried for context. Because E108 was the primary skeletal-muscle annotation, these two states are discussed in Section 2.4 where relevant, rather than listed as separate columns in Table 1. Table 2 provides the direction and significance summaries for the two cross-cohort datasets, GSE268211 and GSE213363.

### 4.10. Region-Level Differential Methylation Analysis

To complement the locus-level screening, we performed region-level differential methylation detection using the EPICv1 mode of the DMRcate R package (https://bioconductor.org/packages/DMRcate/, accessed on 21 September 2026). We applied this to the complete preprocessed probe pool, a 195 × 368,596 β matrix, with HIIT vs. control as the contrast; the region-level analysis used the full pool rather than the variance-ranked subset described in Section 4.2, so that regional smoothing operated on contiguous genomic coverage. Per-CpG moderated t statistics were obtained from a linear model with the design~binary_class + study_group, testing the HIIT-versus-control coefficient (coef = 2) with sub-cohort as a covariate and calling individual CpGs at the package default per-CpG false-discovery rate of 0.05. Adjacent statistics were then smoothed with a Gaussian kernel (lambda = 1000 bp, C = 2), regions were required to contain at least two CpGs, and per-CpG statistics were spatially combined across neighboring probes using Stouffer’s method [47]. Each identified differentially methylated region (DMR) reported its chromosomal coordinates, span, CpG count, region mean methylation difference, and HMFDR. Region-level significance is reported using the harmonic-mean-*p* false-discovery rate (HMFDR < 0.05) as the primary criterion, with the Stouffer-combined *p* < 0.05 threshold retained as a descriptive statistic. As a sensitivity analysis, we refitted the regional model with sex included as an additional covariate (DMRcate version 2.16.1; R version 4.3.3). Because two of the three sub-cohorts were male-only, the sex effect was estimated largely from the Inter1 sub-cohort. We therefore used this analysis to check whether the same regions were recovered at consistent coordinates after sex adjustment, rather than to draw further inference from changes in region-level significance. The specific DMR counts, top region list, and biological annotation are presented in Results Section 2.3 and Table S2.

We additionally performed a participant-aware regional sensitivity analysis using the genome-wide mixed-model estimates. The β-scale model included binary HIIT status, sub-cohort, and sex as fixed effects and a participant-specific random intercept, fitted by REML to 195 observations from 52 participants. Signed Wald z statistics (HIIT coefficient divided by its standard error) and the corresponding adjusted β differences were supplied to DMRcate 2.16.1 with hg19 coordinates. Of 368,596 CpGs, 368,590 had finite standard errors and *p* values and entered this analysis; 6 were excluded. The CpG-level significance indicators used genome-wide BH-adjusted *p* < 0.05. Regional calling used lambda = 1000 bp, C = 2, min.cpgs = 2 and pcutoff = “fdr”. For this sensitivity analysis, HMFDR denotes the native DMRcate 2.16.1 harmonic mean of the constituent CpGs’ BH-adjusted *p* values; regions were summarized at native HMFDR < 0.05. Region mean Δβ is the mean of the adjusted HIIT coefficients. The participant-aware results are reported separately in Section 2.3 and the additional panel of Table S2.

Furthermore, we mapped the loci from the candidate shortlist to unique named genes. We then intersected these genes with the DMRcate significant set, which yielded an overlap gene list. For each gene in this list, we provided the details of its supporting DMR, including chromosomal coordinates, span, and CpG count. The specific overlap counts and the complete gene list are presented in Section 2.3.

### 4.11. DNAm Age Clock Evaluation

To characterize the effect of the HIIT intervention on the methylation age landscape, we evaluated five published epigenetic age clocks [48]: Horvath 2013 (353 CpG, pan-tissue), Hannum 2013 (71 CpG, blood-derived), Horvath 2018 SkinBlood (391 CpG, blood + skin), PhenoAge [23] (513 CpG, phenotypic-age target), and DunedinPACE [24] (~173 CpG, a pace-of-aging measure derived from longitudinal MRI and biomarkers). For each sample, DNAmAge was computed using each clock’s original coefficients and intercept. Between 34.8% and 60.1% of the CpGs of each clock were present in the preprocessed probe matrix; CpGs absent from the matrix were dropped from the weighted sum without imputation and without renormalization of the remaining weights, and the per-clock coverage is reported in Table S9.

Based on the DNAmAge output, we conducted a two-tier comparative analysis. Epigenetic age acceleration was defined as the residual from a within-cohort regression of DNAm age on chronological age [49]. Positive residuals indicate a DNAm age higher than expected for chronological age, and negative residuals a lower one. The first tier was a whole-cohort comparison of age acceleration under each clock between the HIIT samples (n = 102) and the control samples (n = 93). Because the two groups largely comprised the same participants, this comparison is reported descriptively. The second tier comprised a within-subject paired comparison, restricted to subjects with same-sub-cohort Inter2 baseline and 12-week HIIT samples. This yielded 15 paired subjects, totaling 30 samples that formed 15 baseline-to-12-week pairs. For each clock, we computed the within-subject change in age acceleration (12 weeks − baseline), retaining raw clock-value changes as a sensitivity analysis. We then tested the mean change against zero using paired *t*-tests and Wilcoxon signed-rank tests, applying Benjamini–Hochberg FDR correction across the five clocks [50]. This paired within-participant comparison is the primary analysis for the clock results. The numerical results of this two-tier analysis and the concordance of the 5 clocks are presented in Results Section 2.7.

### 4.12. Code and Data Availability

All raw data resources can be obtained from public databases. The discovery cohort GSE171140 (HIIT skeletal muscle, EPIC v1.0) and three cross-cohort evaluation sets—GSE268211 (partner-laboratory external cohort, EPIC v1.0), GSE213363 (PCOS exercise, EPIC v1.0), and GSE60655 (endurance training, 450 K)—are available for download from NCBI GEO. Processed data, analysis scripts, figure source data, and main output files used to reproduce the main analyses of this paper are hosted in a public GitHub repository (https://github.com/BrewSSS/hiit-dna-methylation-analysis, accessed on 21 September 2026). Exact Python (version 3.9) and R package versions (version 4.3.3), the environment files, the random seeds and the scripts for each major analysis are held in the same repository.

## 5. Conclusions

This study developed and applied a batch-aware, participant-aware candidate-prioritization framework for longitudinal skeletal-muscle DNA methylation data, integrating statistical screening, subject-grouped nested cross-validation, and multi-task consensus analysis. Applied to the HIIT cohort GSE171140, the framework prioritized 21 L6 CpGs, of which 20 remained associated with training status in participant-aware mixed-model analyses and 19 remained significant after BH-FDR correction across all 368,596 analyzed CpGs. The TNXB cluster emerged as the most coherent regional signal, supported by a 25-CpG DMR and same-tissue directional evidence, while loci near SIM1 and PIWIL4 provided additional exploratory leads for molecular follow-up. Cross-cohort evaluations showed the greatest consistency under matched-tissue conditions, with attenuated transfer across tissue and disease contexts. Overall, these findings establish a conservative and reusable framework for prioritizing exercise-associated methylation candidates in small longitudinal cohorts and provide a focused set of loci for subsequent genotype-aware, transcriptomic, and functional investigation.

## Figures and Tables

**Figure 1:**
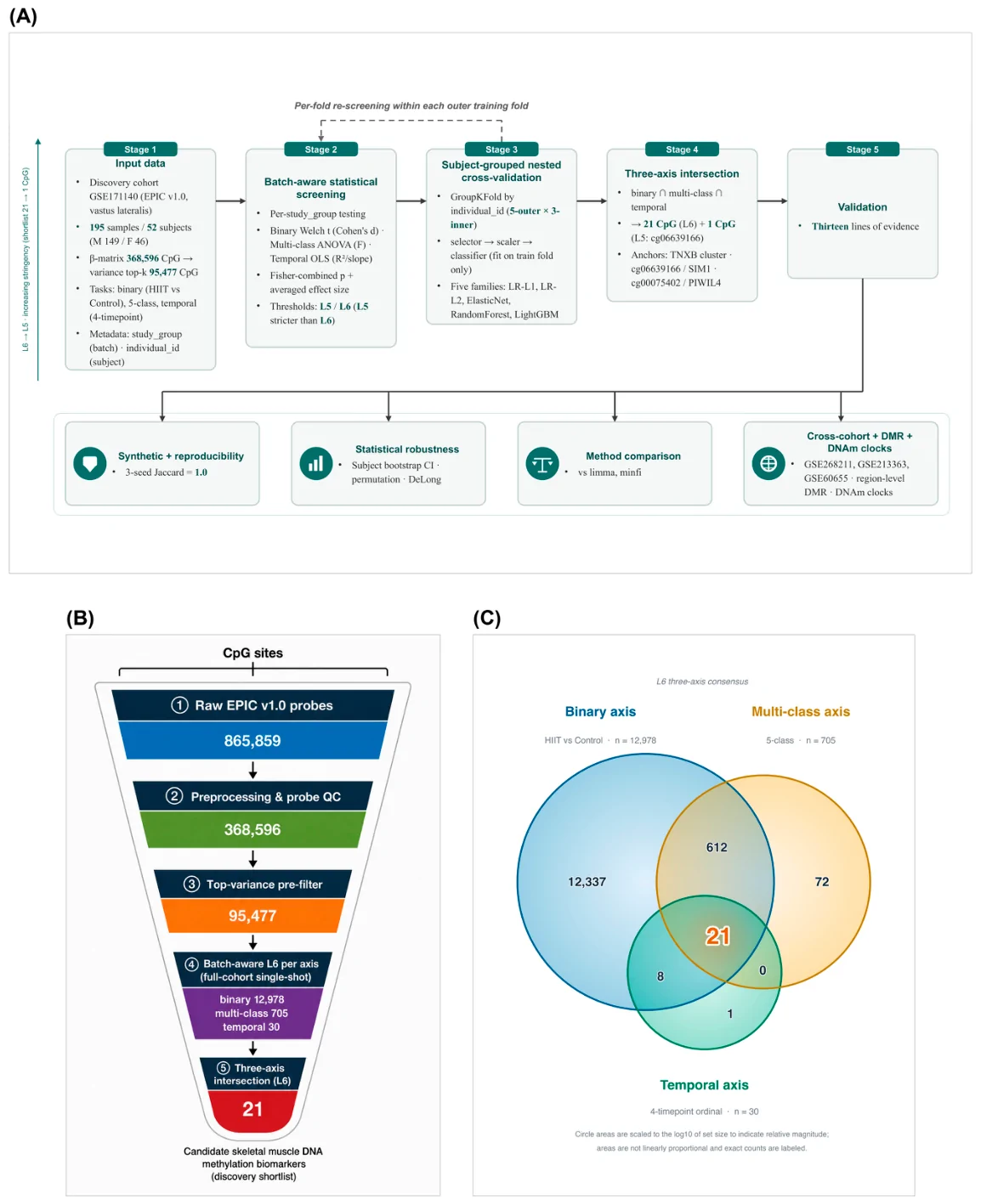
Overview of the batch-aware, subject-grouped candidate-discovery pipeline and the CpG-filtering funnel. (**A**) Pipeline applied to the discovery cohort GSE171140 (195 samples, 52 subjects): per-sub-cohort (study_group) batch-aware screening with Fisher’s-method combination, 5 × 3 subject-grouped nested cross-validation (GroupKFold by individual_id), a binary–multi-class–temporal three-axis consensus, and the evaluation suite (Table S5). (**B**) CpG-filtering funnel: 865,859 raw EPIC v1.0 probes → 368,596 after preprocessing → 95,477 high-variance → 21-CpG three-axis intersection. (**C**) Three-axis Venn of the binary, multi-class and temporal sets; the intersection is the 21-CpG L6 shortlist. CV, cross-validation. In (**A**), the top-variance pre-filter is applied once to the full matrix, whereas the statistical screening within cross-validation is refitted inside each training fold (Section 4.7.5).

**Figure 2:**
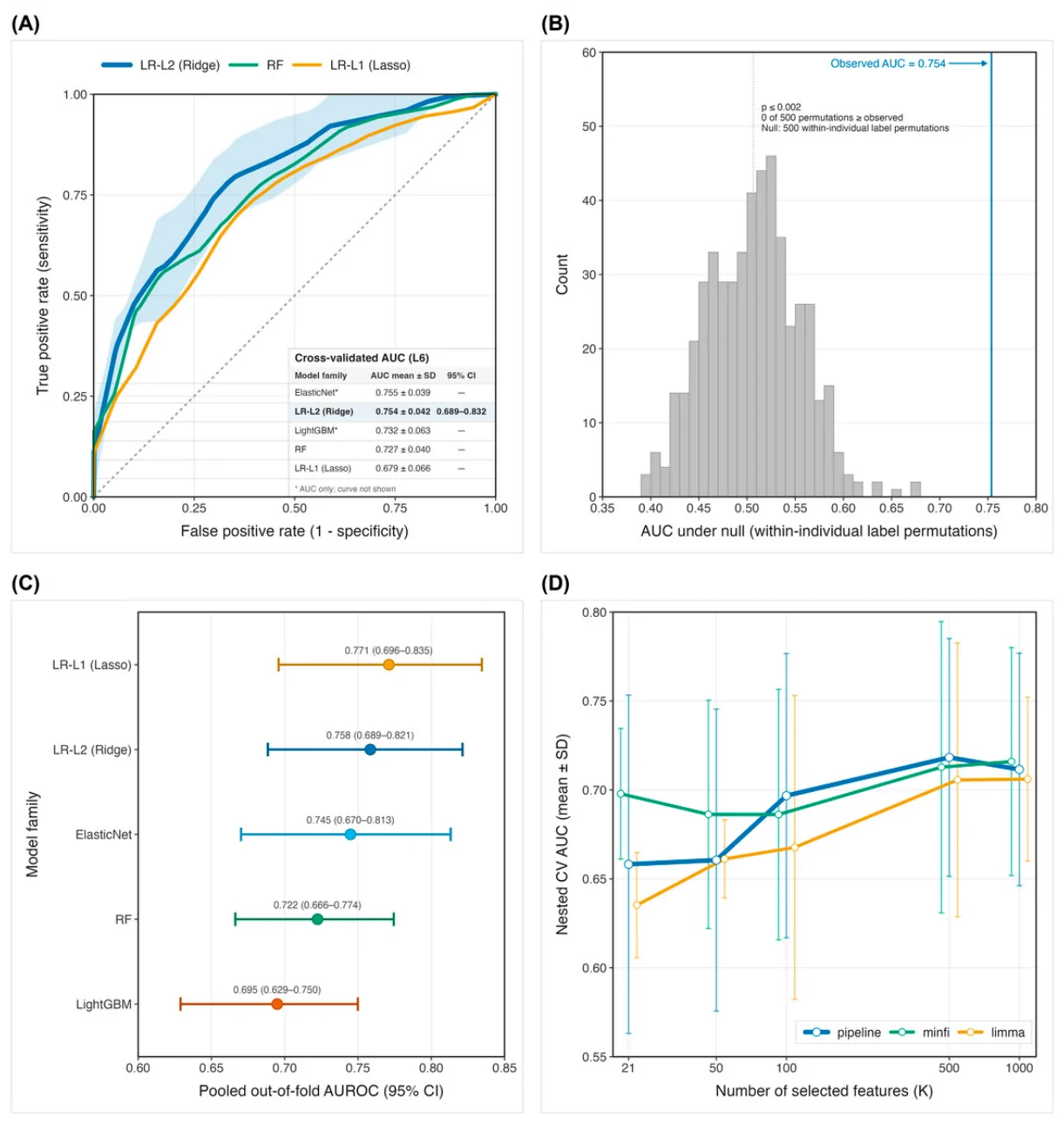
Internal performance and robustness of the binary HIIT vs. Control classifier. (**A**) ROC curves of the binary HIIT vs. control classifier, showing per-fold 95% CI bands and a mean AUC of 0.754. (**B**) Permutation null analysis (500 within-individual shuffles). The observed AUC exceeds the maximum null value (0.676), with a one-sided *p* ≤ 0.002. (**C**) Pooled out-of-fold AUROC with subject-clustered bootstrap 95% CIs for the five model families. (**D**) Method-comparison K-curve (mean AUC ± error band) for the pipeline versus limma and minfi at K = 21–1000. AUC, area under the ROC curve.

**Figure 4:**
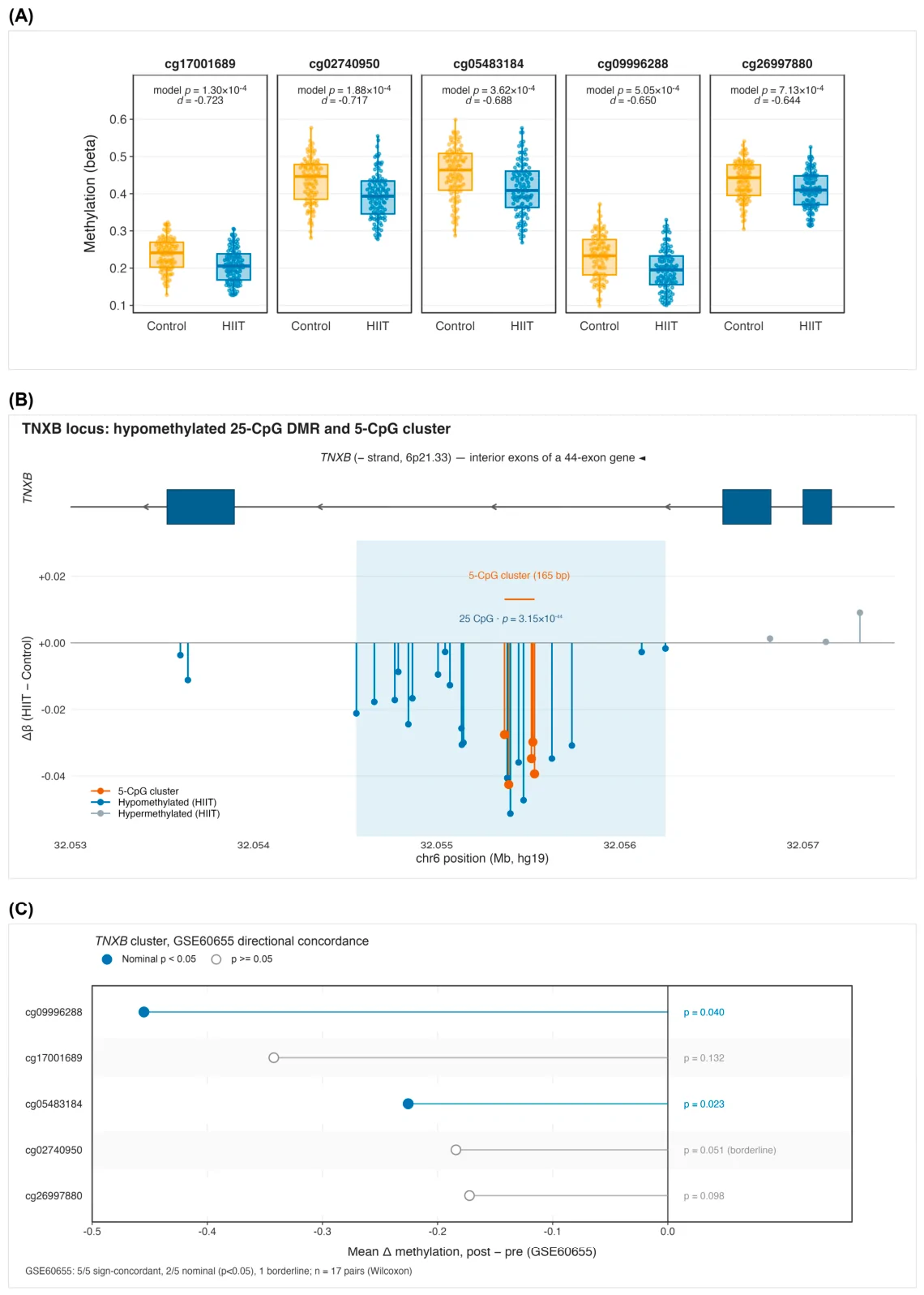
The TNXB five-CpG hypomethylation cluster and same-tissue cross-array directional concordance. (**A**) Beta-value box plots of the five TNXB cluster CpGs in the control (n = 93) versus HIIT (n = 102); boxes show the median and interquartile range (IQR), whiskers extend to 1.5 × IQR, and all individual samples are overlaid as points. (**B**) DMRcate region-level track (hg19) of the chr6:32,054,561–32,056,249 TNXB DMR (25 CpGs, Stouffer *p* = 3.15 × 10^−44^) in chr6p21.33, within the extended MHC. The gene model at the top shows TNXB exons (dark blue boxes) and introns (lines), with arrows indicating the direction of transcription (minus strand). (**C**) Same-tissue cross-array directional concordance in the GSE60655 endurance-training cohort (450 K, vastus lateralis, 34 analytical samples from 17 paired participants), showing per-CpG effect +/− 95% CI. All 5/5 testable TNXB CpGs were direction-concordant, 2/5 were nominally significant, and none survived FDR correction. DMR, differentially methylated region.

**Figure 5:**
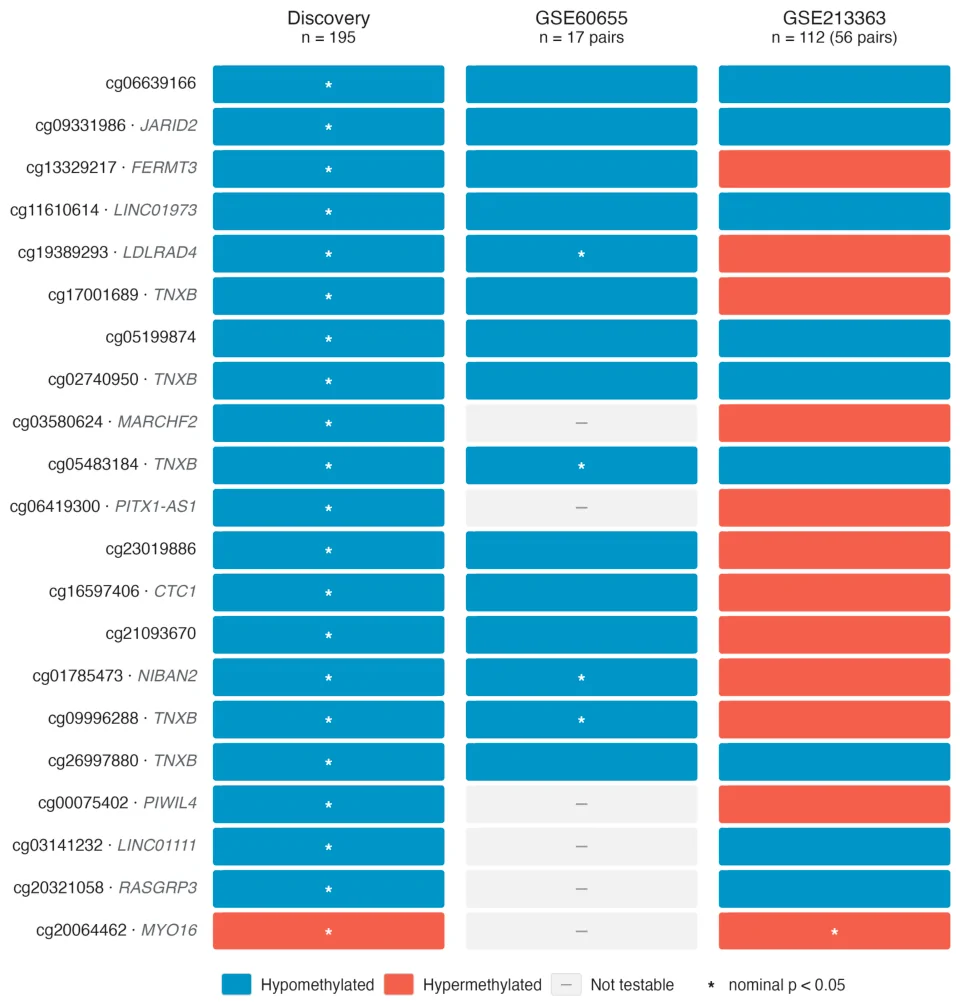
Cross-cohort directional comparison of the 21 candidate CpGs. Heatmap of 21 CpGs (rows) across the discovery cohort (GSE171140) and two external cohorts (columns: GSE60655, endurance-training 450 K same tissue; GSE213363, cross-tissue cross-disease). Cell color indicates effect direction (hypo- vs. hypermethylation), while asterisks denote uncorrected nominal significance. Gray cells represent untestable CpGs in a given cohort. All 21 CpGs were testable in GSE213363 (paired Wilcoxon signed-rank tests across the 56 pre–post pairs). Column labels show sample size (n). In GSE60655, no CpG survived BH-FDR correction; in GSE213363, one CpG (cg20064462) did. GSE268211 is not included due to the unavailability of per-CpG data; its five-model transfer results are summarized in Table 2 as a small partner-laboratory consistency check.

**Figure 6:**
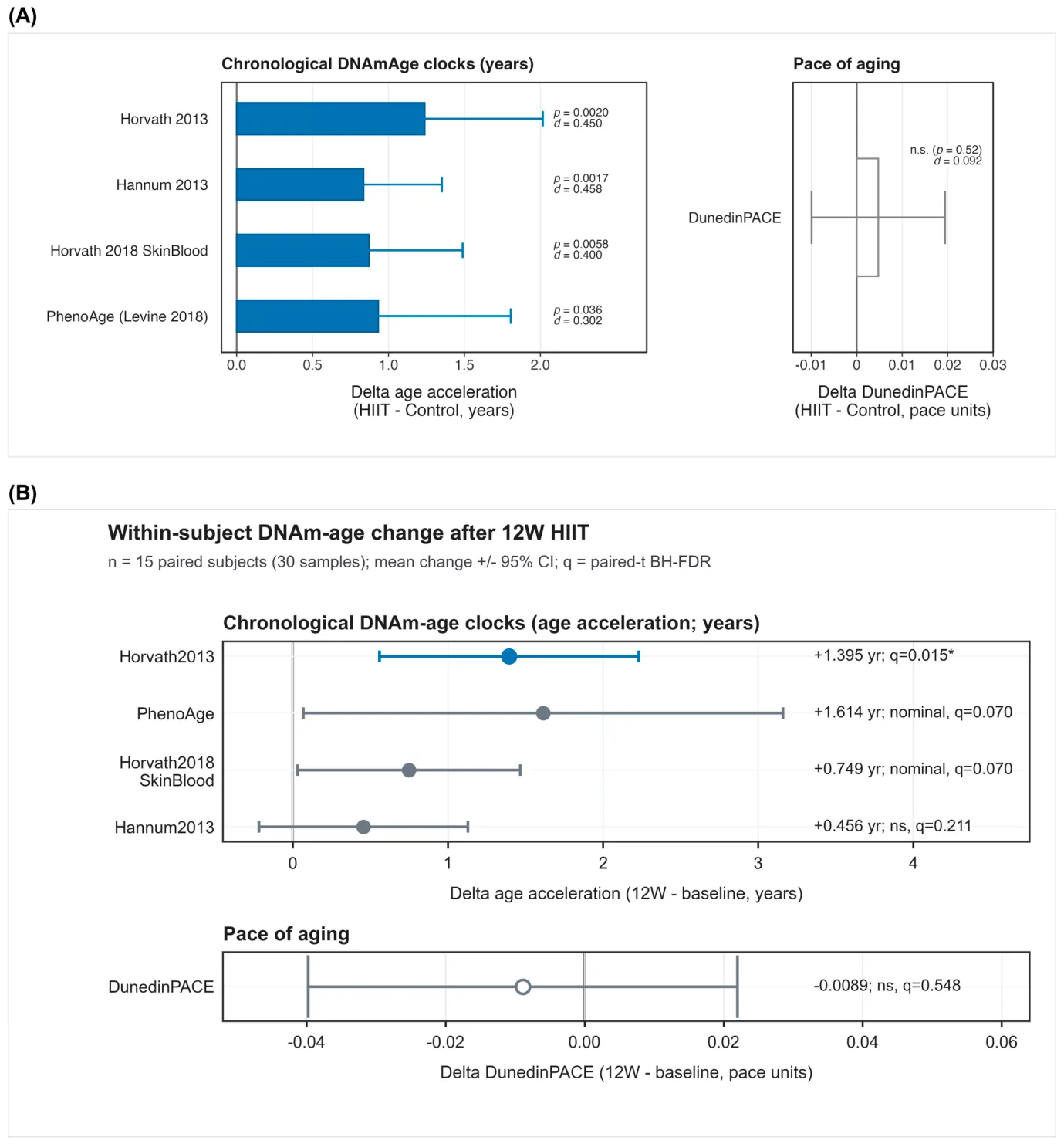
DNA-methylation age-clock response to 12 weeks of HIIT. (**A**) Cross-sectional age acceleration (Δ) ± 95% CI for five DNAm clocks (Horvath 2013 [25], Hannum 2013 [26], Horvath SkinBlood 2018 [27], PhenoAge [23], DunedinPACE) [24]; the four chronological clocks (years) showed higher age acceleration in the HIIT samples (Δ ≈ +0.8 to +1.2 years), whereas the pace-of-aging measure DunedinPACE (pace units) was unchanged. (**B**) Within-subject DNAm-age acceleration change from Inter2 baseline to 12-week HIIT in 15 paired subjects (30 samples), shown as mean Δ ± 95% CI; only Horvath2013 remained significant after BH-FDR across five clocks. In (**B**), q denotes the Benjamini–Hochberg FDR-adjusted paired t-test p value across the five clocks; filled blue dot, q < 0.05 (marked with *); filled grey dots, not significant after FDR correction (nominal, raw p < 0.05 but q ≥ 0.05; ns, raw p ≥ 0.05); open white dot, DunedinPACE (pace-of-aging measure, pace units).

**Table 1:** Annotation of the 21 CpG candidate shortlist.

#	CpG ID	chr:position (hg19)	Gene	Region	CpG Context	d	*p* (Binary)	q (Screen)	q (Mixed Model)	F (Multi)	R^2^ (Temp.)	ChromHMM E108	EWAS Hits	GSE60655 (Sign/Raw *p*)
1	cg20321058†	chr2:33,663,160	RASGRP3	5′UTR	Open sea	−0.465	3.94 × 10^−4^	0.134	0.429	3.28	0.184	5_TxWk	23	not in 450 K pool
2	cg06419300	chr5:134,409,639	PITX1-AS1 (formerly C5orf66)	5′UTR	Open sea	−0.682	3.67 × 10^−4^	0.134	3.95 × 10^−4^	3.45	0.156	15_Quies	3	not in 450 K pool
3	cg09331986	chr6:15,411,400	JARID2	Body	Open sea	−0.830	7.76 × 10^−6^	0.076	1.11 × 10^−4^	5.10	0.180	5_TxWk	10	concordant; *p* = 0.190
4	cg26997880	chr6:32,055,370	TNXB	Body	Island	−0.644	7.13 × 10^−4^	0.136	1.77 × 10^−5^	3.05	0.151	14_ReprPCWk	5	concordant; *p* = 0.098
5	cg05483184	chr6:32,055,393	TNXB	Body	Island	−0.688	3.62 × 10^−4^	0.134	8.09 × 10^−6^	3.04	0.165	14_ReprPCWk	11	concordant; *p* = 0.023
6	cg09996288	chr6:32,055,517	TNXB	Body	Island	−0.650	5.05 × 10^−4^	0.134	8.68 × 10^−6^	3.02	0.152	14_ReprPCWk	5	concordant; *p* = 0.040
7	cg17001689	chr6:32,055,525	TNXB	Body	Island	−0.723	1.30 × 10^−4^	0.120	2.92 × 10^−6^	3.64	0.156	14_ReprPCWk	3	concordant; *p* = 0.132
8	cg02740950	chr6:32,055,534	TNXB	Body	Island	−0.717	1.88 × 10^−4^	0.120	2.82 × 10^−6^	3.29	0.154	14_ReprPCWk	5	concordant; *p* = 0.051
9	cg06639166	chr6:100,913,923	intergenic (near SIM1)	—	N_Shore	−0.921	5.24 × 10^−6^	0.063	9.81 × 10^−5^	6.04	0.200	13_ReprPC	6	concordant; *p* = 0.159
10	cg03141232	chr8:77,362,997	LINC01111	Body	Open sea	−0.567	2.58 × 10^−3^	0.160	0.002	3.04	0.158	15_Quies	5	not in 450 K pool
11	cg01785473	chr9:130,341,161	NIBAN2 (formerly FAM129B)	Body	Open sea	−0.653	1.39 × 10^−3^	0.150	0.003	3.16	0.159	7_Enh	18	concordant; *p* = 0.017
12	cg05199874	chr11:9,623,178	intergenic	—	Open sea	−0.719	9.33 × 10^−4^	0.143	0.005	4.49	0.160	7_Enh	5	concordant; *p* = 0.174
13	cg13329217	chr11:63,975,481	FERMT3	Body	S_Shore	−0.771	2.53 × 10^−4^	0.127	9.07 × 10^−5^	3.44	0.160	1_TssA	2	concordant; *p* = 0.109
14	cg00075402	chr11:94,300,101	PIWIL4	TSS1500	Open sea	−0.586	9.73 × 10^−4^	0.143	0.065	3.91	0.200	15_Quies	0	not in 450 K pool
15	cg21093670	chr11:129,684,147	intergenic	—	N_Shore	−0.653	3.57 × 10^−5^	0.103	0.015	3.88	0.181	15_Quies	25	concordant; *p* = 0.120
16	cg23019886	chr12:6,277,045	intergenic	—	Open sea	−0.681	4.22 × 10^−4^	0.134	0.005	3.52	0.152	7_Enh	50	concordant; *p* = 0.579
17	cg20064462	chr13:109,582,080	MYO16	Body	Open sea	+0.754	1.58 × 10^−4^	0.120	5.15 × 10^−4^	3.21	0.154	15_Quies	0	not in 450 K pool
18	cg16597406	chr17:8,130,173	CTC1 (formerly C17orf68)	3′UTR	S_Shelf	−0.677	6.35 × 10^−4^	0.136	4.32 × 10^−4^	3.14	0.160	1_TssA	20	concordant; *p* = 0.109
19	cg11610614	chr17:75,877,811	LINC01973 (formerly FLJ45079)	Body	Open sea	−0.741	6.18 × 10^−4^	0.136	0.003	3.83	0.154	7_Enh	7	concordant; *p* = 0.243
20	cg19389293	chr18:13,641,872	LDLRAD4 (formerly C18orf1)	Body	Island	−0.730	3.64 × 10^−4^	0.134	7.38 × 10^−5^	3.50	0.166	5_TxWk	59	concordant; *p* = 0.045
21	cg03580624	chr19:8,488,356	MARCHF2 (formerly MARCH2)	Body	Open sea	−0.714	9.73 × 10^−4^	0.143	7.40 × 10^−4^	3.42	0.154	7_Enh	53	not in 450 K pool

**Table 2:** Cross-cohort summary across the discovery dataset and three independent cohorts.

Dataset (GEO)	Lab	Platform	Tissue	n	n Paired	Intervention/Disease	Predictive Result	CpG Concordance	Context
GSE171140 (discovery)	Eynon laboratory (Victoria University)	EPIC	Vastus lateralis	195	52 unique participants	HIIT, 12 weeks	LR-L2 internal CV 0.754 ± 0.046 (95% CI [0.689, 0.832])	Not applicable (discovery cohort)	Twenty-one-CpG shortlist (L6) and one-CpG L5 hit (cg06639166)
GSE268211	Sharples laboratory (Norwegian School of Sport Sciences)	EPIC	Skeletal muscle	10	5 paired	HIIT, baseline vs. 8 weeks	Transfer AUROC 0.96–1.00 across five models; bootstrap 95% CIs wide ([0.75, 1.00]) or degenerate ([1.00, 1.00] for RF and ElasticNet); accuracy 0.50–0.90 (Table S6)	Not assessed (few shortlist CpGs were testable in the external feature set)	Small partner-laboratory consistency check (same platform, same tissue)
GSE213363	Jenkins laboratory (BYU)	EPIC	Whole blood	112	56 paired	Resistance + aerobic; PCOS women	Subject-grouped 5-fold CV 0.673 ± 0.065	21/21 shortlist CpGs testable; paired Wilcoxon: 1/21 BH-FDR < 0.05 (cg20064462, direction-concordant); cg00075402/PIWIL4 nominal only in the unpaired screen (paired *p* = 0.18) (Table S7)	Exploratory cross-context; not replication
GSE60655	Nitert/Lindholm laboratory	450 K	Vastus lateralis	34	17 paired	Endurance training, 3 months	AUROC 0.876 (folds 1.00/0.94/1.00/0.67/0.78)	15/15 sign; 4/15 nominal; 0/15 FDR; TNXB 2/5 nominal	Endurance direction; not HIIT-specific

**Table 3:** Cohort characteristics of the discovery dataset.

Study Group	Time Point	n Samples	n Male	n Female	n Unique Participants	Notes
Main	Baseline	11	11	0	11	Pre-intervention; control class
Main	4-week HIIT	12	12	0	11	HIIT class
Main	Control	12	12	0	11	Non-exercise; control class
Inter1	Baseline	40	20	20	40	Pre-intervention; control class
Inter1	4-week HIIT	40	20	20	40	HIIT class
Inter1	Control	7	1	6	7	Non-exercise; control class
Inter2	Baseline	17	17	0	17	Pre-intervention; control class
Inter2	4-week HIIT	18	18	0	17	HIIT class
Inter2	8-week HIIT	17	17	0	17	HIIT class (Inter2 only)
Inter2	12-week HIIT	15	15	0	15	HIIT class (Inter2 only)
Inter2	Control	6	6	0	6	Non-exercise; control class
Total	All timepoints	195	149	46	52	HIIT n = 102; control n = 93

## Data Availability

The datasets presented in this study are openly available in the NCBI Gene Expression Omnibus (GEO) under accession numbers GSE171140, GSE268211, GSE213363, and GSE60655. The processed data, analysis scripts, and figure source data supporting the findings of this study are available in the GitHub repository at https://github.com/BrewSSS/hiit-dna-methylation-analysis, accessed on 21 September 2026.

## Supplementary Materials

The following supporting information can be downloaded at: https://www.mdpi.com/article/10.3390/ijms27198523/s1.
