# Supplementary material for: A Batch-Aware, Subject-Grouped Pipeline to Prioritize HIIT-Associated DNA Methylation Candidates with Cross-Cohort Evaluation in Skeletal Muscle
Source: Int J Mol Sci. 2026 Sep 24;27(19):8523. doi: 10.3390/ijms27198523 (PMC13665045; doi:10.3390/ijms27198523)
Supplement: Supplementary file 1 [file ijms-27-08523-s001.zip › ijms-4482594-supplementary.pdf]

__Supplementary Material__

1. Supplementary Figures

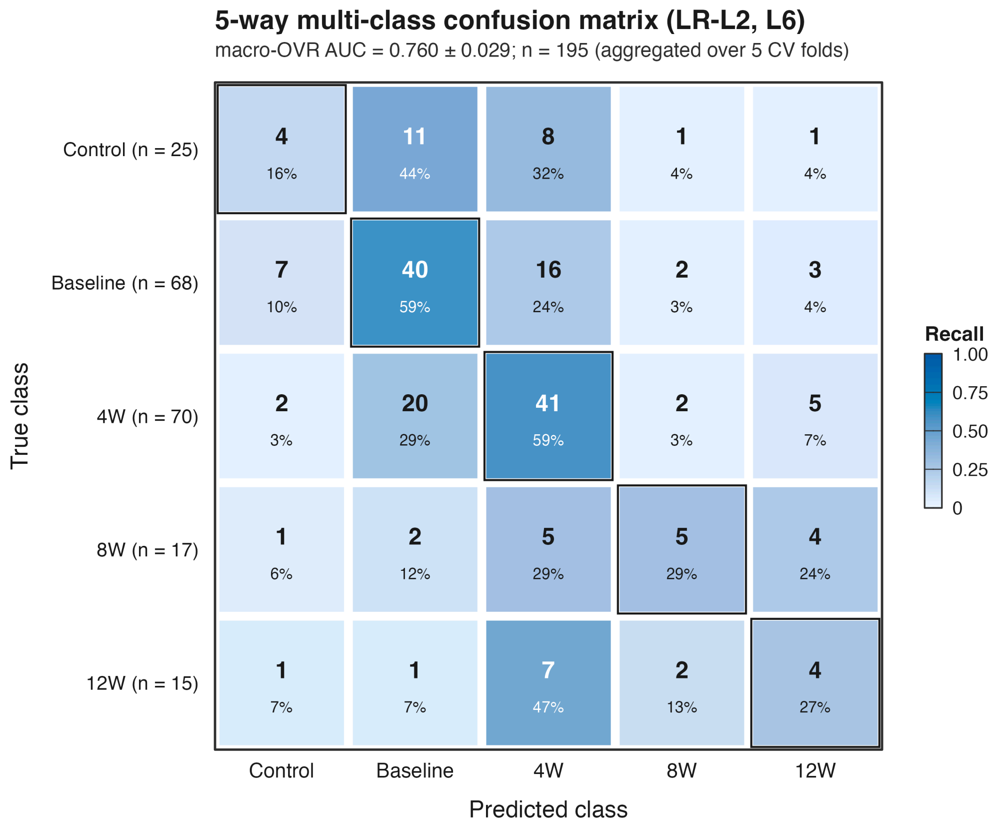

Figure S1. Multi-class five-way confusion matrix (L6 threshold × LR-L2 [Ridge] model). Aggregated confusion matrix from five-fold cross-validation for the multi-class five-way task (Control / Baseline / 4W / 8W / 12W). Rows represent the true class, columns the predicted class, and cell values indicate sample counts. True-class sample counts are Control 25, Baseline 68, 4W 70, 8W 17, and 12W 15; the macro-OVR mean AUC is 0.760 ± 0.029. Comparatively high misclassification between Control and Baseline reflects epigenetic overlap between these two classes within the cohort. 12W is predominantly misclassified as 4W, consistent with the study design in which the 8W and 12W time points occur only in the Inter2 sub-cohort (see Results Section 3.2 and Section 4.7).

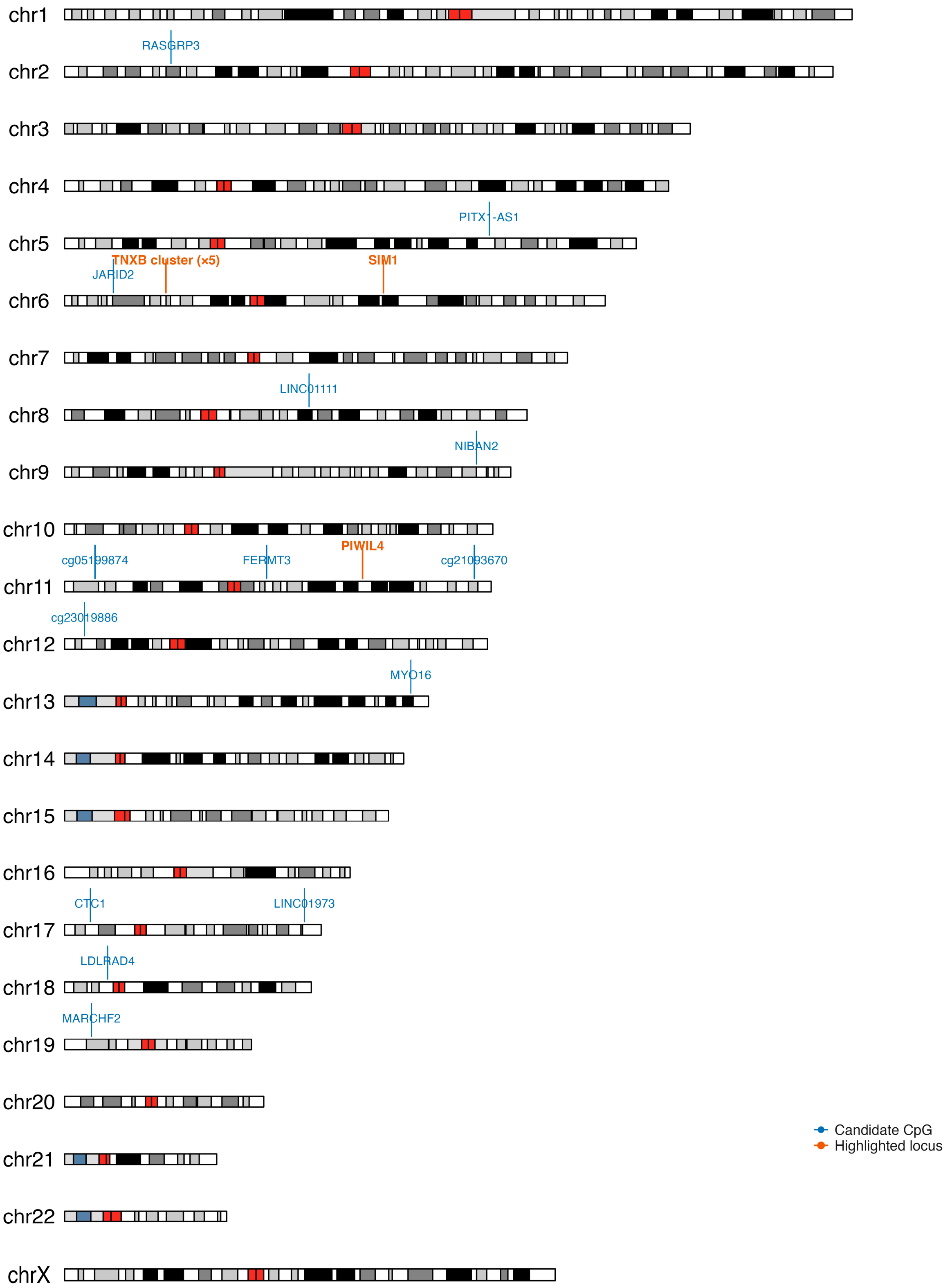

Figure S2. Chromosomal distribution (ideogram) of the 21 candidate CpGs. This ideogram displays the hg19 chromosomal positions of the 21 L6 candidate CpGs, which span 11 chromosomes. Blue ticks mark candidate CpGs, and orange highlights denote the sentinel loci: the TNXB five-CpG cluster, cg06639166 / SIM1, and cg00075402 / PIWIL4. Gene labels use current HGNC symbols. The five CpGs of the TNXB cluster are located within 165 bp and are annotated as a single "TNXB cluster (×5)".

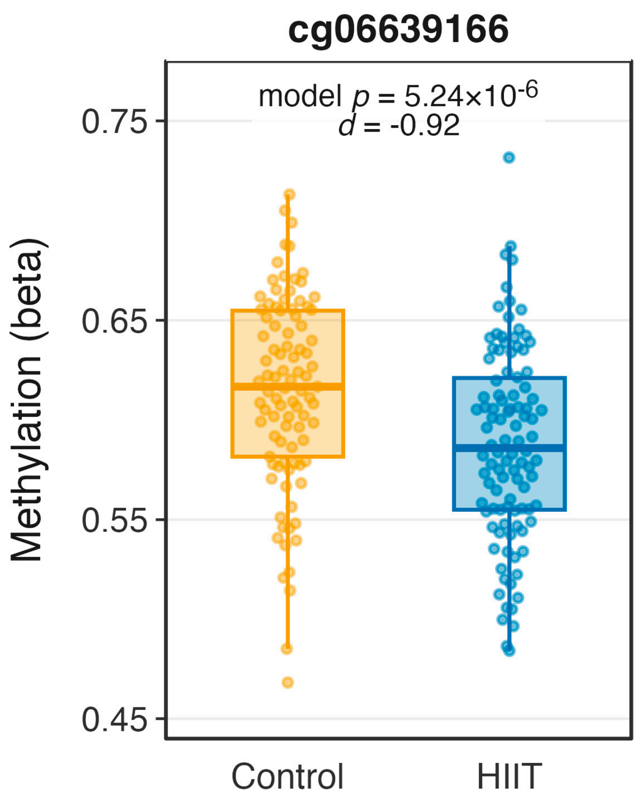

Figure S3. Methylation distribution of cg06639166 by study group. Box plots overlaid with individual data points display the methylation β values of cg06639166 (chr6:100,913,923; approximately 1 kb upstream of the SIM1 transcription start site) in the Control (n = 93) and HIIT (n = 102) groups. The HIIT group exhibits globally lower β values (hypomethylation), consistent with this CpG having the largest effect size among the 21 candidates (model Cohen's d = −0.92; binary p = 5.24 × 10−6). Note: Raw β values are presented for distributional context only and are not covariate-adjusted. The effect size reported in the main text is the batch-stratified averaged Cohen's d from the pipeline.

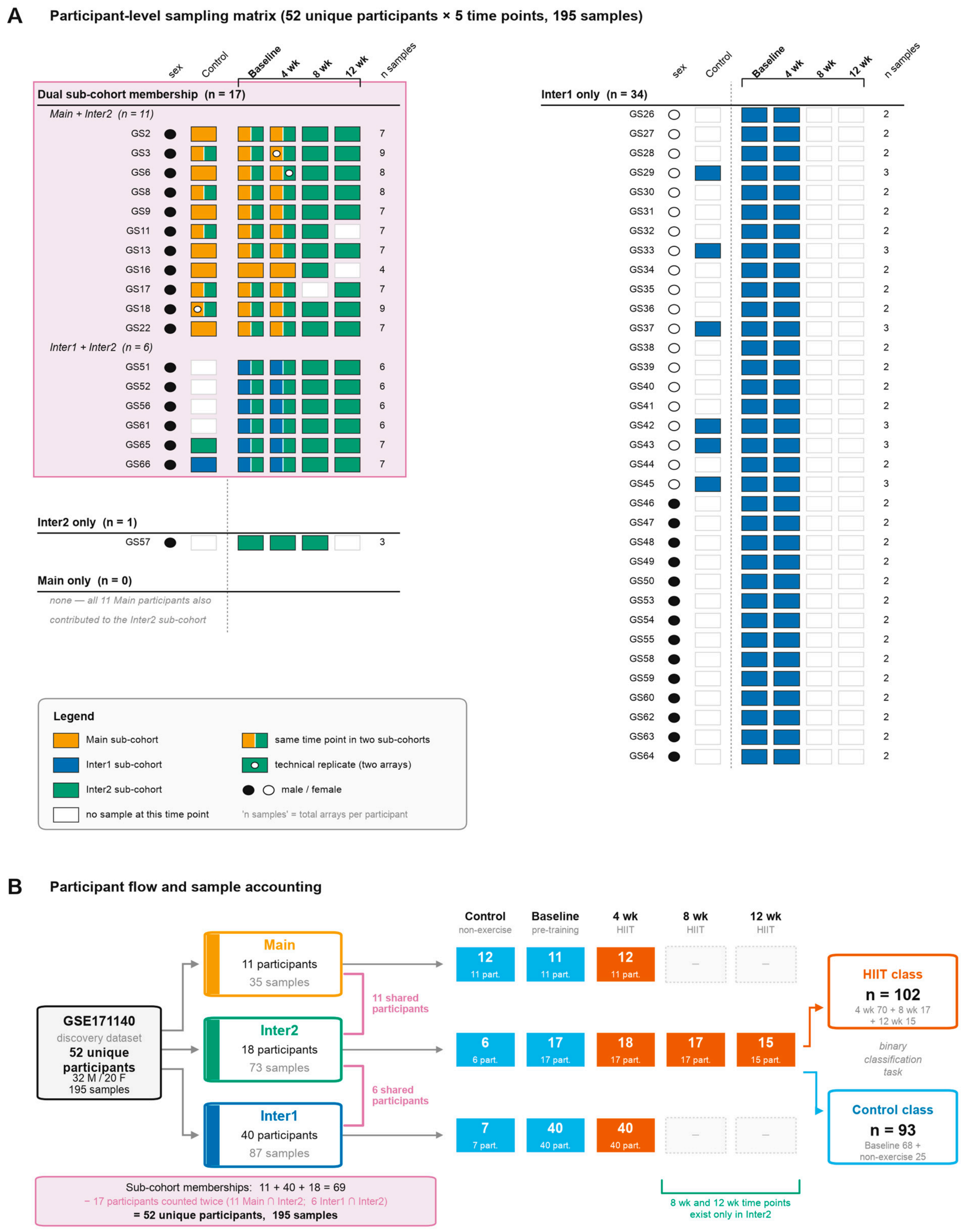

Figure S4. Participant-level sampling structure and participant flow of the GSE171140 discovery dataset. (A) Participant × time-point sampling matrix. Each row is one of the 52 unique participants (identified by individual_id) and each column one of the five sampling occasions: the independent non-exercise Control sampling (left of the dashed rule) and the four longitudinal training time points Baseline, 4 wk, 8 wk and 12 wk (bracketed). Rows are grouped by sub-cohort membership pattern. The 17 participants who contributed to two sub-

cohorts are shown first, inside the tinted band, split into Main ∩ Inter2 (n = 11; GS2, GS3, GS6, GS8, GS9, GS11, GS13, GS16, GS17, GS18, GS22) and Inter1 ∩ Inter2 (n = 6; GS51, GS52, GS56, GS61, GS65, GS66); no participant contributed to Main and Inter1, and none to more than two sub-cohorts. The remaining rows are the single-membership participants (Inter2 only, n = 1; Inter1 only, n = 34; Main only, n = 0, because every Main participant also contributed to Inter2). Cell fill indicates the sub-cohort that provided the sample (orange, Main; blue, Inter1; green, Inter2); a cell divided into two half-width blocks denotes a participant sampled at the same nominal time point under two sub-cohort labels, and the half-widths are categorical, not proportional to sample number. A white dot marks the three cells containing a technical replicate, i.e. two arrays from the same participant, time point and sub-cohort (GS3 4 wk, Main; GS6 4 wk, Inter2; GS18 Control, Main). Empty cells indicate no sample. Sex is shown as a filled (male) or open (female) circle; the right-hand column gives the total number of arrays per participant. The 155 filled cells account for all 195 samples. (B) Participant flow and sample accounting. The 52 unique participants (32 male, 20 female; 195 samples) are distributed across three sub-cohorts whose unique-participant counts sum to 69 because the 17 dual-membership participants (magenta brackets) are counted twice; 69 − 17 = 52. The grid reproduces the per-cell sample counts of Table 3 for every study group × time point, with the number of contributing unique participants given beneath each count; dashed cells indicate combinations that do not exist, showing that the 8 wk and 12 wk time points occur only in Inter2. Grid cells are colored by binary-task class rather than by sub-cohort (vermillion, HIIT class; sky blue, Control class), yielding HIIT n = 102 (4 wk 70 + 8 wk 17 + 12 wk 15) and Control n = 93 (Baseline 68 + non-exercise Control 25). All counts in both panels were derived from the same sample-to-participant mapping used throughout the study and cross-checked against Table 3. Colors follow the colorblind-safe Okabe–Ito palette.

2. Supplementary Tables

Table S1. Synthetic-injection detection benchmark (TPR / injection-defined FDR). This table presents the detection benchmark for known synthetic signals planted in the discovery feature space (195 × 95,477) and recovered through the complete screening funnel (three random seeds: 42 / 123 / 2024). (A) Single-axis (binary) injection: Reports TPR at the binary screening stage for signals planted at Cohen's d ∈ {0.3, 0.5, 0.7, 1.0} (50 CpGs each). (B) Coordinated 3-axis injection: Reports per-axis TPR and intersection injection-defined FDR for signals planted simultaneously across the binary, multi-class, and temporal axes at d ∈ {0.2, 0.3, 0.5, 0.8} (200 coordinated plus 300 single-axis CpGs). The coordinated three-axis intersection TPR reaches only 0.018 at d = 0.8. The intersection set shrinks by approximately 325-fold (from ≈ 45,441 binary L6 candidates to ≈ 140 intersection candidates), indicating that the three-axis intersection is a conservative prioritization procedure, not a high-sensitivity recovery procedure. Note: FDR values in this synthetic benchmark are injection-defined, calculated against the planted ground truth, and are not BH-FDR values from observed discovery-cohort CpG association tests.

A. Single-axis (binary) injection — detection TPR by effect size. Single-axis planted CpGs are recovered 0 times at the three-axis intersection stage because the intersection requires a coordinated three-axis signal. This confirms that the intersection serves as a conservative prioritization rather than a single-axis recovery method.

Level	Effect d	TPR seed42	TPR seed123	TPR seed2024	TPR mean ± SD	FPR mean	n detected (mean)
L5	0.3	0.50	0.76	0.58	0.613 ± 0.133	0.127	47,030

Level	Effect d	TPR seed42	TPR seed123	TPR seed2024	TPR mean ± SD	FPR mean	n detected (mean)
L5	0.5	0.74	0.92	0.82	0.827 ± 0.090	0.127	47,030
L5	0.7	0.92	1.00	0.98	0.967 ± 0.042	0.127	47,030
L5	1.0	1.00	1.00	1.00	1.000 ± 0.000	0.127	47,030
L6	0.3	0.56	0.76	0.60	0.640 ± 0.106	0.155	57,265
L6	0.5	0.80	0.94	0.86	0.867 ± 0.070	0.155	57,265
L6	0.7	0.96	1.00	0.98	0.980 ± 0.020	0.155	57,265
L6	1.0	1.00	1.00	1.00	1.000 ± 0.000	0.155	57,265

B. Coordinated 3-axis injection — per-axis TPR and intersection injection-defined FDR (mean over 3 seeds). The intersection set shrinks by approximately 325× (from ≈ 45,441 binary L6 candidates to ≈ 140 intersection candidates). Coordinated TPR peaks at only 0.018 (d = 0.8), while injection-defined FDR remains high due to single-axis spillover and the null pool. These FDR values are calculated against the planted synthetic ground truth and should not be interpreted as BH-FDR values from the observed discovery-cohort CpG association tests.

Axis / stage	Effect d	TPR mean ± SD	FDR mean	n detected (mean)
Binary	0.2	0.182 ± 0.011	0.999	45,441
Binary	0.3	0.317 ± 0.012	0.998	45,441
Binary	0.5	0.514 ± 0.007	0.997	45,441
Binary	0.8	0.740 ± 0.026	0.995	45,441
Multi-class	0.2	0.050 ± 0.007	0.995	2,975
Multi-class	0.3	0.109 ± 0.007	0.989	2,975
Multi-class	0.5	0.263 ± 0.026	0.973	2,975
Multi-class	0.8	0.532 ± 0.023	0.946	2,975
Temporal	0.2	0.000 ± 0.000	1.000	153
Temporal	0.3	0.000 ± 0.000	1.000	153
Temporal	0.5	0.006 ± 0.004	0.989	153
Temporal	0.8	0.012 ± 0.007	0.976	153
3-axis intersection (coordinated)	0.2	0.000 ± 0.000	1.000	140
3-axis intersection (coordinated)	0.3	0.000 ± 0.000	1.000	140
3-axis intersection (coordinated)	0.5	0.007 ± 0.008	0.991	140

Axis / stage	Effect d	TPR mean ± SD	FDR mean	n detected (mean)
3-axis intersection (coordinated)	0.8	0.018 ± 0.010	0.974	140

Table S2. Top 30 differentially methylated regions (DMRs), HIIT versus Control. This table lists the top 30 DMRs, identified by DMRcate (EPIC v1 mode; Stouffer combined $p < 0.05$) in the discovery cohort GSE171140 (hg19) and ranked by Stouffer p. Columns include chromosomal coordinates, region width (bp), number of CpGs, Stouffer p, HMFDR, region mean $\Delta\beta$, and overlapping gene(s) as annotated by DMRcate. A negative mean $\Delta\beta$ indicates hypomethylation with HIIT. TNXB (rank 3, 25 CpGs) and SIM1 (rank 7, 17 CpGs; rank 15, 15 CpGs) provide region-level support for the sentinel loci of this study. An additional panel below reports the four focus regions recovered in the participant-aware sensitivity analysis (Section 2.10); its native DMRcate 2.16.1 HMFDR values and adjusted mean $\Delta\beta$ are reported separately from the original top-30 analysis above.

#	Chr	Start	End	Width (bp)	CpGs	Stouffer p	HMFDR	mean $\Delta\beta$	Overlapping gene(s)
1	chr3	147,122,315	147,127,193	4,879	37	2.62×10^{-56}	8.59×10^{-4}	−0.0141	ZIC1, ZIC4
2	chr2	223,164,459	223,169,609	5,151	28	2.44×10^{-50}	6.32×10^{-4}	−0.0218	CCDC140
3	chr6	32,054,561	32,056,249	1,689	25	3.15×10^{-44}	4.22×10^{-4}	−0.0246	TNXB
4	chr10	102,994,605	102,998,762	4,158	21	5.65×10^{-42}	1.13×10^{-3}	−0.0172	LBX1-AS1
5	chr6	30,649,909	30,653,799	3,891	27	1.70×10^{-41}	1.98×10^{-3}	−0.0181	PPP1R18
6	chr14	60,972,853	60,978,852	6,000	25	8.81×10^{-37}	7.18×10^{-4}	−0.0169	SIX6, C14orf39
7	chr6	100,903,561	100,906,895	3,335	17	8.25×10^{-36}	9.05×10^{-4}	−0.0207	SIM1
8	chr4	174,427,146	174,432,425	5,280	22	4.92×10^{-35}	5.83×10^{-4}	−0.0142	—
9	chr10	102,976,327	102,979,058	2,732	18	6.52×10^{-35}	6.43×10^{-4}	−0.0197	—
10	chr1	119,530,932	119,532,925	1,994	16	3.51×10^{-34}	5.67×10^{-4}	−0.0198	TBX15
11	chr6	33,244,775	33,247,166	2,392	44	1.35×10^{-31}	2.94×10^{-3}	−0.0104	B3GALT4, WDR46
12	chr3	147,104,488	147,109,629	5,142	19	5.94×10^{-30}	2.42×10^{-3}	−0.0164	ZIC4-AS1, ZIC4
13	chr2	66,671,478	66,673,985	2,508	15	4.77×10^{-29}	4.12×10^{-4}	−0.0160	MEIS1
14	chr8	145,727,318	145,729,799	2,482	19	1.66×10^{-28}	1.69×10^{-3}	+0.0155	PPP1R16A, GPT, CTD-2517M22.14
15	chr6	100,894,513	100,897,445	2,933	15	1.13×10^{-27}	1.57×10^{-3}	−0.0175	SIM1
16	chr3	35,719,509	35,721,758	2,250	17	2.56×10^{-27}	3.81×10^{-4}	+0.0195	ARPP21
17	chr19	35,632,493	35,634,556	2,064	12	2.56×10^{-27}	4.44×10^{-4}	−0.0169	FXYD1, CTD-2527I21.4, FXYD7, LGI4
18	chr19	2,250,901	2,253,854	2,954	16	7.38×10^{-27}	1.64×10^{-3}	−0.0163	AMH, JSRP1

#	Chr	Start	End	Width (bp)	CpGs	Stouffer p	HMFDR	mean Δβ	Overlapping gene(s)
19	chr1	221,053,841	221,055,964	2,124	17	1.29×10−25	2.35×10−3	−0.0177	HLX
20	chr3	158,390,329	158,390,821	493	11	1.42×10−25	4.44×10−4	−0.0183	GFM1, LXN
21	chr19	46,282,571	46,286,475	3,905	16	5.98×10−25	6.02×10−4	−0.0053	DMPK, AC011530.4, DMWD
22	chr4	111,549,880	111,553,018	3,139	15	9.45×10−25	1.97×10−3	−0.0211	PITX2
23	chr6	30,698,584	30,698,987	404	11	1.09×10−24	3.07×10−4	−0.0164	FLOT1
24	chr16	68,269,295	68,271,177	1,883	16	4.06×10−23	1.09×10−3	−0.0145	ESRP2, RP11-96D1.6
25	chr1	217,309,906	217,312,060	2,155	16	4.06×10−23	4.83×10−4	−0.0182	ESRRG
26	chr11	65,406,254	65,408,496	2,243	11	6.67×10−23	1.13×10−3	−0.0163	SIPA1
27	chr21	38,067,101	38,070,671	3,571	18	1.20×10−22	3.07×10−3	−0.0149	—
28	chr21	38,076,709	38,080,274	3,566	15	2.31×10−22	2.16×10−3	−0.0172	SIM2
29	chr4	8,200,261	8,202,708	2,448	13	2.48×10−22	1.57×10−3	−0.0167	SH3TC1
30	chr19	35,629,022	35,630,651	1,630	16	5.39×10−22	1.78×10−3	+0.0137	FXYD1, CTD-2527I21.4, LGI4, AC020907.2

Table S2 (additional panel). Participant-aware regional sensitivity analysis: four focus regions (hg19).

Focus region	Chr	Start (hg19)	End (hg19)	CpGs	Native HMFDR	Mean Δβ
TNXB	chr6	32,053,600	32,056,820	28	2.09E-05	−0.02233
SIM1 body 1	chr6	100,903,561	100,906,895	17	5.11E-04	−0.01896
SIM1 body 2	chr6	100,893,665	100,897,445	16	5.35E-03	−0.01548
cg06639166 region	chr6	100,913,923	100,915,805	8	6.24E-04	−0.01980

Native HMFDR is the harmonic mean of the genome-wide BH-adjusted CpG p values within each region in DMRcate 2.16.1. Mean Δβ averages the participant-aware model's adjusted HIIT coefficients; negative values indicate hypomethylation with HIIT. The recovered intervals cover the corresponding original regions; identical boundaries were not required.

Table S3. Variance-structure analysis of the discovery feature space. This table presents a PERMANOVA-style variance partition (Euclidean distance; 999 permutations) of the discovery feature space (195 samples × 95,477 high-variance CpGs), attributing the total multivariate variance to each factor (R^2 = SS_between / SS_total).

Factor	Levels	R^2 (variance explained)	Pseudo-F	Permutation p
individual_id (subject)	52	0.624	—	— (nested)
study_group (sub-cohort / batch)	3	0.195	23.21	0.001
sex	2	0.086	18.15	0.001
time (5-class)	5	0.041	2.02	0.001
HIIT vs. Control	2	0.015	3.01	0.001

As a 52-level nested factor, the subject factor (individual_id) was not amenable to a permutation test. Its dominance, however, was independently corroborated by the within-/between-subject distance ratio, where the mean within-subject squared distance was 0.56 × the between-subject value. Principal component analysis (PCA) revealed no single dominant axis, with PC1 accounting for 17.6% and PC2 for 12.7% of the total variance. Study_group explained 88.9% of PC1, and individual_id explained 65.5% of PC2. In contrast, training duration (time) explained only 7.3% of PC1 and 4.9% of PC2. Consequently, subject identity and sub-cohort/batch emerged as the dominant variance components. This finding motivated the adoption of a subject-grouped, batch-aware design. Seed = 42.

Table S4. Extended-MHC / mQTL / CNV / ancestry / probe-risk annotation of the five TNXB CpGs. This table provides annotations for the five TNXB CpGs, including CpG ID, hg19 coordinate, TNXB/DMR membership, extended-MHC status, discovery direction, and directional concordance with the GSE60655 endurance-training cohort. It also indicates whether mQTL, CNV, ancestry/population-stratification, or SNP-affected-probe contributions could be directly assessed. Due to the limitations of available methylation-only public data, these genetic effects could not be directly tested and thus cannot be excluded. None of the five TNXB CpGs appears on published lists of cross-reactive or SNP-affected EPIC probes (Section 3.4). The five CpGs are located within a 165 bp segment (chr6:32,055,370–32,055,534) of a CpG island (UCSC hg19 chr6:32,055,067–32,055,601, 534 bp). They also belong to the TNXB DMR (chr6:32,054,561–32,056,249), which contains 25 CpGs and shows a Stouffer p = 3.15 × 10^-44 and HMFDR = 4.22 × 10^-4. All five CpGs are hypomethylated with HIIT. While direction-concordant, they are FDR-non-significant (0/5) in the GSE60655 endurance cohort. Thus, the signal is best characterized as an internally coherent candidate regional methylation signal within the extended MHC (6p21.33, MHC class III; RCCX segmental-duplication module), rather than as validated HIIT-specific TNXB or extracellular-matrix biology.

CpG	hg19 (chr6)	Discovery (HIIT vs Control)	GSE60655 endurance concordance
cg26997880	32,055,370	hypomethylation ($p = 7.13 \times 10^{-4}$)	concordant, hypo (p = 0.098, ns)
cg05483184	32,055,393	hypomethylation ($p = 3.62 \times 10^{-4}$)	concordant, hypo (p = 0.023, nominal)
cg09996288	32,055,517	hypomethylation ($p = 5.05 \times 10^{-4}$)	concordant, hypo (p = 0.040, nominal)
cg17001689	32,055,525	hypomethylation ($p = 1.30 \times 10^{-4}$)	concordant, hypo (p = 0.132, ns)
cg02740950	32,055,534	hypomethylation ($p = 1.88 \times 10^{-4}$)	concordant, hypo (p = 0.051, borderline)

Table S5. Pipeline evaluation portfolio: thirteen lines of evidence

#	Category	Evidence	Numeric anchor	Source	Figure	Section
1	Internal CV	LR-L2 binary AUROC, subject-grouped nested CV	0.754 ± 0.046; 95% CI [0.689, 0.832]	Internal CV analysis	Fig. 2A	3.1
2	Statistical robustness	Permutation null test (500 within-individual iterations)	p ≤ 0.002 (observed 0.754; max null 0.676)	Permutation analysis	Fig. 2B	3.1
3	Statistical robustness	Subject-level bootstrap 95% CI (1000 replicates)	[0.689, 0.832]	Bootstrap analysis	Fig. 2A	3.1
4	Statistical robustness	Pooled out-of-fold model comparison (subject-clustered bootstrap)	LR-L2 vs LR-L1 p = 0.225; both logistic models vs LightGBM p ≤ 0.023 (2,000 subject-clustered bootstrap replicates)	Pairwise AUROC comparison	Fig. 2C	3.2
5	Synthetic benchmark	Single-axis binary TPR at d = 0.5	0.83 – 0.87 (L5 / L6 across 3 seeds)	Synthetic injection benchmark	Supp. material	3.1
6	Reproducibility	Three-seed Jaccard index (L5 / L6 sets)	Jaccard = 1.000 across seeds 42, 123, 2024	Reproducibility analysis	—	3.1
7	Method comparison	Comparative AUROC across K values; proposed pipeline ranked highest at K = 100 – 500	K = 21: pipeline 0.658, minfi 0.698, limma 0.635; K = 500: pipeline 0.718, limma 0.706, minfi 0.713	Method comparison analysis	Fig. 2D	3.1
8	External transfer	GSE268211 (n = 10, partner laboratory, EPIC, muscle) — five-model transfer	AUROC 0.96–1.00; CIs wide or degenerate; accuracy 0.50–0.90 (Table S6)	External transfer analysis	— (Table 2)	3.5
9	Cross-cohort	GSE213363 (n = 112, blood, EPIC) — subject-grouped 5-fold CV	0.673 ± 0.065 (per-fold range 0.58 – 0.74)	Cross-disease cohort analysis	Fig. 5	3.5
10	Cross-array	GSE60655 (34 analytical samples; 17 paired participants; 450K; vastus lateralis)	AUROC 0.876; 15 of 15 testable CpGs sign-concordant; 4 of 15 nominally significant; 0 of 15 FDR-significant; endurance	Cross-array same-tissue concordance analysis	Figs. 4C, 5	3.5

#	Category	Evidence	Numeric anchor	Source	Figure	Section
			cohort, not HIIT-specific			
11	Sex sensitivity	Male-only subset and sex-as-covariate (dual evidence)	AUROC ≈ baseline (sex covariate Δ ≤ 0.01; male-only Δ ≤ 0.02 in same direction; sex coefficient ≈ 0)	Sex sensitivity analysis	—	3.6
12	Region-level	DMRcate detection of the TNXB cluster (25 CpGs spanning 1.7 kb) Participant-aware sensitivity analysis recovered TNXB (28 CpGs).	Stouffer p = 3.15×10^{-44}; HMFDR = 4.2×10^{-4}; 8 of 13 shortlist genes overlap a DMR (62%) Participant-aware TNXB: native HMFDR = 2.09×10^{-5}; mean adjusted Δβ = −0.02233.	DMR region analysis; Table S2 (additional panel)	Fig. 4B	3.3
13	DNAm age	Paired within-participant DNAm-age analysis (15 pairs); whole-cohort comparison reported descriptively	Paired within-participant analysis (15 pairs): only Horvath2013 remained significant after BH-FDR (Δ = +1.40 yr, q = 0.015); DunedinPACE null	DNAm age clock analysis	Fig. 6A	3.7

Internal cross-validation was performed using subject-grouped 5-outer × 3-inner nested cross-validation on the discovery dataset (n = 195). External transfer denotes training on the full discovery dataset and prediction on an independent cohort. Model-family comparison used pooled out-of-fold predictions resampled by participant (2,000 bootstrap replicates; Section 2.7.3). AUROC, area under the receiver operating characteristic curve; CI, confidence interval; TPR, true positive rate; HMFDR, harmonic-mean false discovery rate; SD, standard deviation.

Table S6. Five-model external transfer performance on GSE268211.

Model	Transfer AUROC	Bootstrap 95% CI	Accuracy
LR-L1	0.960	[0.760, 1.000]	0.50
LR-L2	0.960	[0.750, 1.000]	0.90
RF	1.000	[1.000, 1.000]	0.90
ElasticNet	1.000	[1.000, 1.000]	0.70
LightGBM	0.960	[0.760, 1.000]	0.80

Models were fitted on the complete discovery cohort (GSE171140, n = 195) and applied once to GSE268211 (n = 10 samples; five paired participants; 44,784 CpGs shared with the discovery feature set). Confidence intervals are 95% bootstrap intervals (1,000 resamples); with five paired participants the intervals are wide or degenerate, and these results are interpreted as a small partner-laboratory consistency check rather than external validation.

Table S7. Paired testing of all 21 shortlist CpGs in GSE213363.

CpG	Gene	Discovery direction	Δβ (post − pre)	95% CI	Wilcoxon p	BH-FDR	Concordant
cg20064462	MYO16	hyper (up)	0.0300	[0.0143, 0.0489]	0.000755	0.0158	Yes
cg03580624	MARCHF2	hypo (down)	0.0135	[-0.0074, 0.0323]	0.0503	0.528	No
cg23019886	intergenic	hypo (down)	0.0066	[-0.0023, 0.0156]	0.119	0.625	No
cg20321058	RASGRP3	hypo (down)	-0.0191	[-0.0375, -0.0029]	0.133	0.625	Yes
cg09996288	TNXB	hypo (down)	0.0009	[-0.0027, 0.0039]	0.149	0.625	No
cg00075402	PIWIL4	hypo (down)	0.0377	[0.0042, 0.0756]	0.184	0.63	No
cg13329217	FERMT3	hypo (down)	0.0023	[-0.0008, 0.0056]	0.221	0.63	No
cg17001689	TNXB	hypo (down)	0.0014	[-0.0013, 0.0041]	0.24	0.63	No
cg05199874	intergenic	hypo (down)	-0.0043	[-0.0106, 0.0020]	0.328	0.765	Yes
cg01785473	NIBAN2	hypo (down)	0.0032	[-0.0052, 0.0114]	0.392	0.819	No
cg19389293	LDLRAD4	hypo (down)	0.0049	[-0.0115, 0.0215]	0.429	0.819	No
cg16597406	CTC1	hypo (down)	0.0041	[-0.0071, 0.0144]	0.568	0.889	No
cg11610614	LINC01973	hypo (down)	-0.0005	[-0.0054, 0.0041]	0.59	0.889	Yes
cg09331986	JARID2	hypo (down)	-0.0080	[-0.0296, 0.0124]	0.683	0.889	Yes
cg06419300	PITX1-AS1	hypo (down)	0.0034	[-0.0176, 0.0236]	0.707	0.889	No
cg21093670	intergenic	hypo (down)	0.0044	[-0.0234, 0.0339]	0.714	0.889	No
cg05483184	TNXB	hypo (down)	-0.0017	[-0.0071, 0.0033]	0.72	0.889	Yes
cg26997880	TNXB	hypo (down)	-0.0010	[-0.0079, 0.0060]	0.788	0.919	Yes
cg02740950	TNXB	hypo (down)	-0.0009	[-0.0055, 0.0038]	0.858	0.935	Yes
cg06639166	intergenic (near SIM1)	hypo (down)	-0.0025	[-0.0134, 0.0088]	0.929	0.935	Yes
cg03141232	LINC01111	hypo (down)	-0.0004	[-0.0107, 0.0090]	0.935	0.935	Yes

Paired two-sided Wilcoxon signed-rank tests across 56 pre–post pairs; Δβ, mean post−pre difference with participant-level bootstrap 95% CI (2,000 resamples); BH-FDR across the 21 tested CpGs. Paired t-tests gave consistent conclusions (three nominal associations, one below the BH-FDR threshold).

Table S8. Direction and paired-test summary for the 15 testable shortlist CpGs in GSE60655.

CpG	Direction	ΔM (post − pre)	95% CI	Wilcoxon p	BH-FDR	Hedges g (paired)	Rank-biserial r
cg01785473	hypo (down)	-0.163	[-0.290, -0.050]	0.0174	0.152	-0.604	-0.647
cg05483184	hypo (down)	-0.226	[-0.381, -0.083]	0.0232	0.152	-0.639	-0.621

CpG	Direction	ΔM (post − pre)	95% CI	Wilcoxon p	BH-FDR	Hedges g (paired)	Rank-biserial r
cg09996288	hypo (down)	-0.455	[-0.807, -0.128]	0.0395	0.152	-0.585	-0.569
cg19389293	hypo (down)	-0.174	[-0.319, -0.025]	0.0448	0.152	-0.524	-0.556
cg02740950	hypo (down)	-0.184	[-0.332, -0.043]	0.0505	0.152	-0.564	-0.542
cg26997880	hypo (down)	-0.172	[-0.337, -0.010]	0.0984	0.199	-0.449	-0.464
cg13329217	hypo (down)	-0.136	[-0.263, -0.011]	0.109	0.199	-0.467	-0.451
cg16597406	hypo (down)	-0.272	[-0.549, 0.007]	0.109	0.199	-0.433	-0.451
cg21093670	hypo (down)	-0.184	[-0.393, 0.038]	0.12	0.199	-0.381	-0.438
cg17001689	hypo (down)	-0.342	[-0.625, -0.052]	0.132	0.199	-0.512	-0.425
cg06639166	hypo (down)	-0.160	[-0.371, 0.052]	0.159	0.217	-0.327	-0.399
cg05199874	hypo (down)	-0.094	[-0.218, 0.018]	0.174	0.218	-0.358	-0.386
cg09331986	hypo (down)	-0.142	[-0.354, 0.094]	0.19	0.219	-0.278	-0.373
cg11610614	hypo (down)	-0.123	[-0.265, 0.007]	0.243	0.261	-0.392	-0.333
cg23019886	hypo (down)	-0.092	[-0.386, 0.201]	0.579	0.579	-0.142	-0.163

Exact two-sided Wilcoxon signed-rank tests across 17 pre–post pairs (the prespecified primary test, identical to the published analysis); paired t-tests as sensitivity gave 6/15 nominal associations (versus 4/15) with no CpG below the BH-FDR threshold under either test. ΔM, mean post−pre difference on the M-value scale with participant-level bootstrap 95% CI (2,000 resamples). For the one participant with technical replicates, the first-batch array pair was used, matching the published analysis.

Table S9. CpG coverage of the five DNAm age clocks on the preprocessed probe matrix.

Clock	CpGs in model	Present on array	Coverage (%)
Horvath 2013	353	123	34.8
Hannum 2013	71	32	45.1
Horvath 2018 SkinBlood	391	223	57.0
PhenoAge	513	203	39.6
DunedinPACE	173	104	60.1

Coverage is the number of clock CpGs present in the 195 × 368,596 preprocessed β-value matrix. Clock CpGs absent from the matrix did not contribute to the weighted sum; they were neither imputed nor replaced, and the weights of the remaining CpGs were not renormalized. DNAm age values from all five clocks are therefore affected by incomplete coverage.
